# Two-dimensional bond-selective fluorescence spectroscopy: violations of the resonance condition, vibrational cooling rate dispersion, and super-multiplex imaging

**DOI:** 10.1039/d5sc02628h

**Published:** 2025-07-30

**Authors:** Philip A. Kocheril, Jiajun Du, Haomin Wang, Ryan E. Leighton, Dongkwan Lee, Ziguang Yang, Noor Naji, Adrian Colazo, Lu Wei

**Affiliations:** a Division of Chemistry and Chemical Engineering, California Institute of Technology Pasadena CA 91125 USA lwei@caltech.edu

## Abstract

Multidimensional spectroscopies have shaped our understanding of molecular phenomena, but they are often limited in sensitivity. In this work, we describe two-dimensional bond-selective fluorescence-detected infrared-excited (2D-BonFIRE) spectro-microscopy: an ultrasensitive two-dimensional spectroscopy and hyperspectral imaging technique. 2D-BonFIRE spectra are richly detailed, allowing for direct measurement of vibronic coupling and strong evidence of combination modes in congested spectral regions. Additionally, 2D-BonFIRE provides new insights into the nature of vibrational relaxation, including direct experimental observation of vibrational cooling rate dispersion, illuminating the inherent heterogeneity of vibrational decays in large molecules. Finally, we demonstrate that the high specificity of 2D-BonFIRE allows for single-shot 16-colour chemical imaging, with high promise for further palette expansion. 2D-BonFIRE holds significant potential as a tool for fundamental photophysics and a basis for super-multiplex bioimaging.

## Abstract 2

2D-BonFIRE (two-dimensional bond-selective fluorescence-detected infrared-excited) spectro-microscopy encompasses detailed molecular spectroscopy, ultrafast vibrational dynamics, and 16-color super-multiplex imaging.

## Graphical abstract

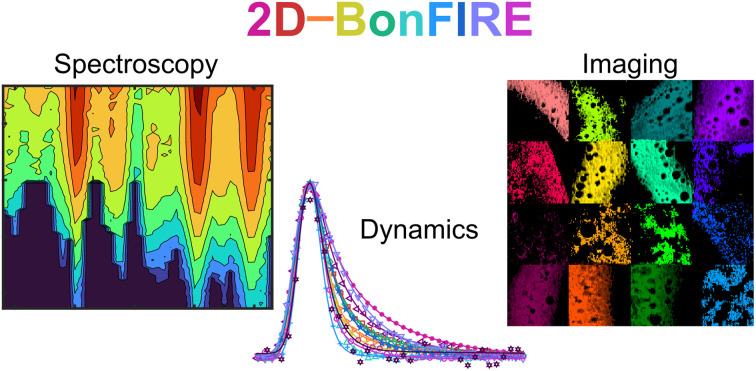

## Introduction

Nonlinear multidimensional spectroscopies have revolutionized our understanding of dynamics in solids and liquids.^1^ The development of two-dimensional (2D) spectroscopies was strongly inspired by magnetic resonance techniques, applying the design principles of radiofrequency pulse sequences to ultrashort optical pulses.^2,3^ 2D optical spectroscopies have proven transformative over the past 25 years in many fields,^4–9^ including biophysics,^10–12^ chemical dynamics,^13^ and energy transport.^14,15^ For example, to probe rich molecular vibrations, 2D infrared (IR) spectroscopy, using a timed sequence of ultrashort IR laser pulses, has provided fundamentally new understandings about vibrational couplings,^16,17^ structural dynamics,^18^ energy transfer pathways,^19,20^ and solvation effects from small molecules to large structural and functional proteins.^21,22^

As absorptive or coherent echo measurements, conventional 2D spectroscopies generally require optically thick samples. For instance, 2DIR measurements on small molecules commonly occur in the 0.1–5 M range.^23,24^ For strongly absorbing probes such as transition metal carbonyls and proteins (which benefit from the presence of many IR-absorbing chemical bonds per single protein), sub-mM measurements may be performed, but measurements at the low μM–nM level remain challenging.^22,25^ Additionally, 2DIR-based microscopies may require long acquisition times (*e.g.*, >6 hours) and face challenges in resolving subcellular details while operating at the diffraction limit.^26^ Thus, a new and complementary method that can probe rich 2D vibrational information ultrasensitively (ideally, at the single-molecule level) is desired for applications in live cells, where analytes are often limited to low concentrations (nM) and relatively fast acquisition times and low peak powers are needed. Such capability combining rich spectral information, high sensitivity, and decent biocompatibility should enable new opportunities for reporting local cellular environments^27^ and facilitate super-multiplex imaging,^28^ thanks to the physically interpretable spectra^29^ and intrinsically narrow linewidths of vibrational spectroscopy.^30^

**Fig. 1:**
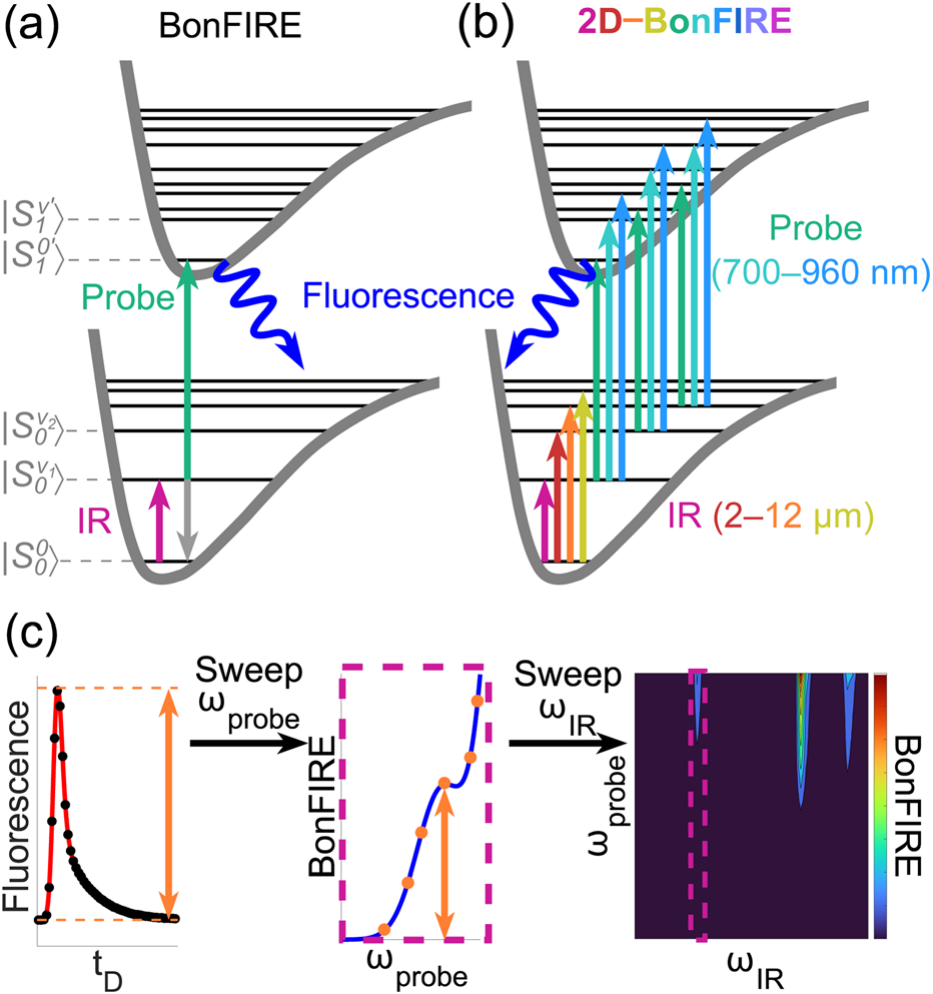
Overview of 2D-BonFIRE. (a) Principle of BonFIRE, comprising two narrowband excitations. Vibronic states are labelled |S^v^_*n*_〉, where *n* describes the electronic state and v describes the vibrational state. Vibrational states are labelled with an apostrophe (') in the S_1_ manifold. (b) Principle of 2D-BonFIRE, where narrowband pulses are tuned across broad frequency ranges. (c) Picosecond-scanning methodology of 2D-BonFIRE. 2D spectra are constructed from a series of 1D sweeps, where BonFIRE intensity is measured by the peak height in a sweep of the time delay (*t*_D_) between the IR and probe pulses.

One scheme for sensitively probing vibrations is vibrational-electronic (vibronic) double-resonance fluorescence spectroscopy (Fig. 1a), as pioneered by Kaiser and co-workers.^31^ Here, a mid-IR pulse excites a vibrational mode in a fluorescent molecule. The vibrationally excited population is then electronically excited by a probe pulse, up-converting it to the S_1_ electronic manifold, from which it can fluoresce back to the ground state. Such mid-IR-based vibronic fluorescence has seen a resurgence in recent years, first revisited by Tokmakoff and co-workers with femtosecond pulses toward single-molecule vibrational spectroscopy in solution.^23,24,32–37^ Our laboratory further reported bond-selective fluorescence-detected infrared-excited (BonFIRE) spectro-microscopy (Fig. 1a),^38^ a live-cell-compatible vibronic fluorescence technique, where narrow vibrational bands are excited efficiently by picosecond pulses due to their high power-per-mode (1.6 ± 0.2 ps; 10 cm^−1^ bandwidth). In recent work, we have demonstrated BonFIRE's single-molecule sensitivity,^39^ wide-field bioimaging capabilities,^40^ and local electric field sensing through vibrational lifetimes, as confirmed by traditional solvatochromism and first-principles calculations.^41^ Several other groups have also explored similar strategies for a variety of applications in the materials and life sciences,^42–48^ demonstrating the power of vibronic fluorescence in modern spectro-microscopy.

Similar to linear Fourier transform IR (FTIR) spectroscopy, these previous reports of vibronic fluorescence were generally one-dimensional (1D) in nature (collectively taken as “1DVF”), primarily focusing on the IR frequency (*ω*_IR_)-dependence of the fluorescent signal and providing detailed vibrational information, such as identifying molecular dimerization under single-molecule conditions.^39^ However, the probe frequency (*ω*_probe_)-dependence remains underexplored, particularly in the high-energy limit. Thus, from the current body of 1DVF literature, one overarching question is largely unanswered: does tuning *ω*_probe_ provide useful 2D information for broader functional applications in both spectroscopy and bioimaging?

To answer this question, we report 2D-BonFIRE: two-dimensional vibronic fluorescence spectroscopy and imaging with mode-selective excitation (Fig. 1b). In the language of 2D spectroscopies, 2D-BonFIRE is an off-diagonal action spectroscopy most comparable to 2D vibrational-electronic (2DVE) spectroscopy,^49,50^ where changes in the absorption of a vibrational pump and electronic probe are inferred through changes in integrated fluorescent intensity. We demonstrate that 2D-BonFIRE allows richly detailed vibronic spectroscopy, revealing strong evidence of previously unknown combination modes in the CH-stretching region and illuminating the inherent heterogeneity of vibrational relaxation. Additionally, we show that 2D-BonFIRE allows fluorophores with highly overlapping spectra to be differentiated, culminating in proof-of-concept demonstrations of single-shot 16-colour chemical imaging (to our knowledge, setting the record) and vibrational lifetime multiplex imaging, with high potential for further expansion.

## Results and discussion

### 2D-BonFIRE spectroscopy in the frequency domain

As illustrated in Fig. 1c (Fig. S1 and SI Section S1),^51–58^ the methodology of 2D-BonFIRE is different from modern 2D spectroscopies based on coherent echoes,^17^ but bears a strong resemblance to the first reported 2D optical spectroscopies (*i.e.*, frequency-resolved pump-probe),^4,8^ including early doubly-resonant sum-frequency generation spectroscopies^59^ and four-wave-mixing spectroscopies,^60^ adopting a picosecond-scanning strategy. For a given *ω*_IR_ and *ω*_probe_, we sweep the time delay between our two pulses, where the height of the peak in fluorescent intensity is taken as true BonFIRE signal (Fig. 1c, left). Leaving *ω*_IR_ fixed, we sweep *ω*_probe_, measuring BonFIRE at each probe frequency (Fig. 1c, middle). We repeat this process for each desired value of *ω*_IR_, allowing us to stitch together a full 2D map (Fig. 1c, right). Our broad tunability in *ω*_IR_ (830–5000 cm^−1^; equivalent to 2–12 μm) and *ω*_probe_ (10 420–14 290 cm^−1^; equivalent to 700–960 nm) allows us to acquire ultra-broadband 2D spectra while remaining immune to the effects of multimode coherences.^35,36^ Importantly, we assume that the fluorescence emission profile does not shift on the picosecond timescale, which is reasonable under Kasha's rule, as we discussed in recent work.^41^ We also note that Zheng and co-workers have explored the possibility of ultrafast shifts in fluorescence emission under similar conditions and observed differences only at very high concentrations (10 mM),^46^ suggesting that the fluorescence emission profile should change negligibly for our current experiments at the μM level.

**Fig. 2:**
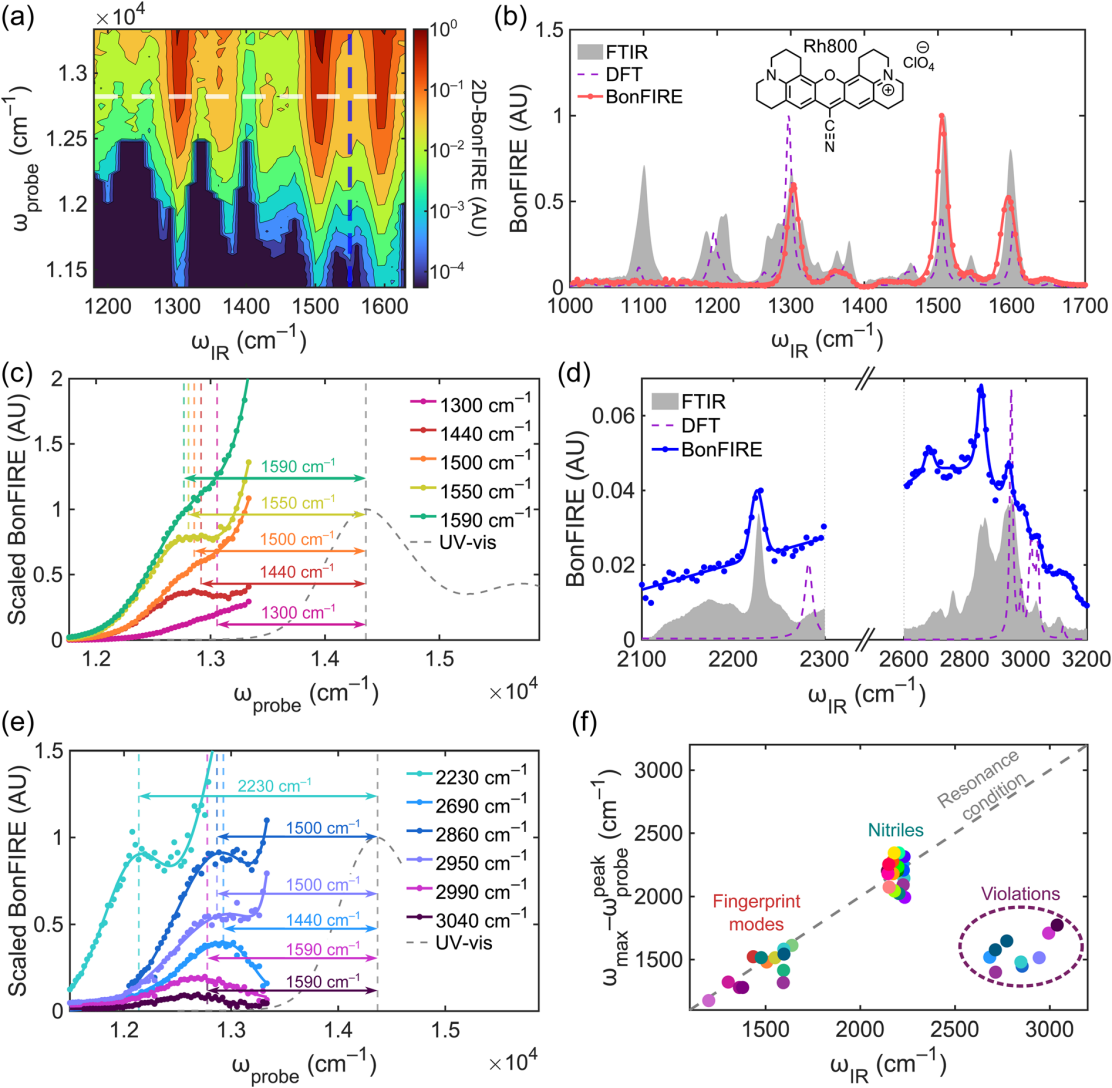
2D-BonFIRE frequency-domain spectroscopy. (a) 2D-BonFIRE fingerprint spectrum of Rh800 at 100 μM in DMSO-d_6_. (b) 2D-BonFIRE intensity as a function of *ω*_IR_ at *ω*_probe_ = 12 820 cm^−1^ (Rh800 structure inset). 2D-BonFIRE aligns well with the FTIR spectrum (grey, 100 mM in DMSO) and the predicted IR absorption by DFT (purple; scaled by 0.975). We note that the absence of certain peaks in BonFIRE compared to FTIR is due to solvent interference from DMSO and dye aggregation due to the high concentrations needed for FTIR, as we have discussed previously.^38,40^ (c) Relative 2D-BonFIRE intensity as a function of *ω*_probe_ for Rh800 at *ω*_IR_ = 1300 (pink), 1440 (red), 1500 (orange), 1550 (yellow) and 1590 (green) cm^−1^. Because these signals vary over orders of magnitude, the spectra have been scaled to facilitate a visual comparison. Vertical dashed line positions are defined relative to the UV-vis absorption maximum. (d) 2D-BonFIRE of Rh800 in the nitrile and CH-stretching regions at *ω*_probe_ = 12 120 cm^−1^. Due to strong anharmonicity, the frequency of the nitrile-stretch is over-estimated in harmonic DFT. The CH-stretching absorptions predicted by DFT (purple; scaled by 0.975) disagree with the FTIR (grey) and 2D-BonFIRE (blue) spectra. (e) Scaled 2D-BonFIRE *ω*_probe_-dependence of Rh800 for the high-frequency modes of Rh800. Vertical dashed line positions are defined relative to the UV-vis absorption maximum. The spectra with *ω*_IR_ > 2600 cm^−1^ are displaced by a different energy than *ω*_IR_, in violation of the resonance condition. (f) Summary of 2D-BonFIRE frequency-domain data for all measured modes in this work (also see Table S2). The resonance condition is well-maintained for the fingerprint modes and nitriles, but the peaks in the CH-stretching region consistently violate the resonance condition. As detailed below, these modes appear to be combination modes involving the simultaneous excitation of two fingerprint modes, where one mode is FC-coupled and the other is not. In such a case, only the FC-coupled mode should contribute to double-resonance.

A 2D-BonFIRE spectrum (Fig. 2a) is first achieved for a well-benchmarked molecule, rhodamine 800 (Rh800, Fig. 2b),^38–41^ in the molecular fingerprint region (∼800–1800 cm^−1^). The fingerprint 2D-BonFIRE spectrum of Rh800 displays a complex response with BonFIRE intensity varying over nearly five orders of magnitude, where the *x*-axis denotes the *ω*_IR_-dependence of BonFIRE signal, the *y*-axis denotes the *ω*_probe_-dependence, and colours closer to red denote higher BonFIRE intensity (Fig. 2a).

The horizontal white dashed line in Fig. 2a represents a 1D-BonFIRE spectrum at a fixed *ω*_probe_ in the pre-resonance excitation regime (Fig. 2b), which generally matches well with both FTIR and density functional theory (DFT; also see Table S1). The differences in relative intensity between BonFIRE and FTIR stem from the Franck–Condon (FC) factors (*f*_FC_) of the excited vibrational modes, which describe the vibronic coupling strength of a given mode.^37^ The combination of strong IR absorptions (*σ*_IR_ ∼ 4000 M^−1^ cm^−1^) and large *f*_FC_ (∼0.04, where |*f*_FC_|^2^ is directly proportional to signal and reaches up to ∼40% of the |*f*_FC_|^2^ of the 0–0 transition, which describes one-photon absorption)^61,62^ leads to efficient BonFIRE in the molecular fingerprint region. Moving the white dashed line in Fig. 2a up or down would allow us to examine the relative vibronic intensity changes across the different vibrational modes.

For a given vibrational mode, the 1D projection along the vertical axis yields a vibronic spectrum that resembles the ultraviolet-visible (UV-vis) electronic absorption spectrum of the dye (Fig. 2a and c). For example, the blue dashed line in Fig. 2a corresponds to the yellow curve in Fig. 2c with *ω*_IR_ fixed at 1550 cm^−1^ (number indicated in the legend in the upper-right corner in Fig. 2c). This profile, in the region of *ω*_probe_ + *ω*_IR_ ≤ *ω*_max_ (UV-vis absorption maximum, *ω*_max_ = 14 370 cm^−1^ or *λ*_max_ = 696 nm for Rh800 in DMSO^41^), is found to be redshifted by 1550 cm^−1^ (number shown between the peaks of the yellow BonFIRE and grey UV-vis spectra), exactly matching the energy of the absorbed IR photon, as expected. This indicates rigorous double-resonance excitation, defined as the “resonance condition” in vibronic fluorescence.^34,63^

Similarly, we next examined the *ω*_probe_-dependence of four other strong fingerprint modes from our 2D-BonFIRE spectrum, pictured by moving the blue dashed line in Fig. 2a to the left or right (Fig. 2c; note that signal intensities were scaled for visual comparison). All the fingerprint modes were found to obey the resonance condition, exhibiting profiles redshifted by the corresponding IR excitation energy. We do observe differences in the sharpness of the peak in the *ω*_probe_-domain between different modes (Fig. 2c), with some modes showing clearer peaks (1440 and 1550 cm^−1^) and other modes exhibiting saddle-like peaks (1300, 1500, and 1590 cm^−1^). We do not fully understand these subtle lineshape differences at this time, but we note that they may be related to molecular symmetry (see Section S2).

Furthermore, the 2D-BonFIRE spectra are found to deviate from the resonance condition in the region of *ω*_probe_ + *ω*_IR_ > *ω*_max_ (Fig. 2c, regions to the right of the vertical, coloured dashed lines), exhibiting far larger intensities than expected. In this region, *ω*_probe_ is of sufficiently high energy to excite molecules from S^v^_0_ to a range of possible states in the S_1_ manifold, as well as being able to excite from a range of vibrational states in the S_0_ manifold to 
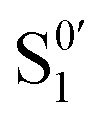
 (Section S2 and Fig. S2). As such, we rationalize that 2D-BonFIRE in the *ω*_probe_ + *ω*_IR_ > *ω*_max_ regime is best understood from the perspective of multi-state-to-multi-state transitions, where the total fluorescent intensity increases due to multiple distinct transitions being excited simultaneously (where even if the individual cross-sections do not exceed that of *ω*_probe_ + *ω*_IR_ = *ω*_max_, the sum of cross-sections does). We reason that coherent oscillations are not observed, despite the suspected involvement of multiple states, because the initial and final states are different (and therefore distinguishable), while indistinguishability is a requirement for multimode coherences.^24^ To validate the generality of this interpretation, we tested several other popular commercial dyes (sulfo-cyanine 5.5 (Cy5.5), ATTO665, ATTO680, ATTO725, magnesium phthalocyanine, and deuterated indocyanine green (ICG-d_7_)), confirming the trends we observed with Rh800 (Fig. S3).

We next explored 2D-BonFIRE in the cell-silent region (Fig. 2d; 1800–2300 cm^−1^), a key region for vibrational bioimaging due to minimal background from endogenous biomolecules.^41^ When present in large organic dyes, nitrile-stretching vibrations appear in the cell-silent region and can maintain large *f*_FC_ (∼0.06) but tend to have small *σ*_IR_ (∼30 M^−1^ cm^−1^).^38^ Consistent with the fingerprint modes, the nitrile-stretch of Rh800 was found to obey the resonance condition (Fig. 2e, cyan, 2230 cm^−1^). A slanted baseline exists across the cell-silent region, which we now conclude to originate from non-degenerate resonance-enhanced two-photon absorption (NDR-TPA),^36^ or the simultaneous absorption of *ω*_IR_ + *ω*_probe_, where the cross-section is enhanced due to the photons being near-resonant for the dye (Section S3 and Fig. S4).^64^

### 2D-BonFIRE identifies violations of the resonance condition

We then moved to examine Rh800 in the CH-stretching region (∼2600–3200 cm^−1^; Fig. 2d), which has remained relatively underexplored in previous 1DVF works. In contrast to nitriles, CH-stretching bands tend to have stronger *σ*_IR_ (∼500 M^−1^ cm^−1^) but vanishingly small *f*_FC_ (∼10^−6^).^37,65^ Surprisingly, the 2D-BonFIRE peaks in the CH-stretching region break the trend established in the nitrile and fingerprint regions. Despite being excited with IR photon energies above 2600 cm^−1^ and the BonFIRE peak positions agreeing very well with FTIR (Fig. 2d), the 2D-BonFIRE spectra of all five major modes show profiles only redshifted by 1400–1600 cm^−1^ from the UV-vis spectrum (Fig. 2e), in clear violation of the resonance condition. To our knowledge, this is the first such observation in vibronic fluorescence spectroscopies. This result is particularly surprising in the context of Kaiser and co-workers' early measurements of the NH-stretching vibration in coumarin 7.^63^ The NH-stretch presents similarly to the CH-stretches (high *σ*_IR_, low *f*_FC_), but was observed to maintain the resonance condition.^63^

Upon close inspection, we see that the redshifts agree very well with three of the fundamental modes that we measured in the fingerprint region (1440, 1500, and 1590 cm^−1^; Fig. 2c). These redshifts suggest that up-conversion is occurring from these fingerprint states, despite the IR photons not being resonant with those fundamental modes. The question then becomes: how does the vibrational energy arrive at those fingerprint modes?

One possible theory is that the IR-excited state is a CH-stretch, but it subsequently decays into a BonFIRE-active fingerprint mode, which was reported by Sakai in IR-UV double-resonance in the gas phase.^66^ In such a case, the rise-time of the signal should be delayed by ∼1 ps,^66^ due to the requisite time for the exchange of vibrational energy. However, the rise-times exhibit no delay in 2D-BonFIRE (Section S4 and Fig. S5a), ruling out this hypothesis. We note that we have shown previously that our system can resolve vibrational lifetime dynamics as short as 0.2 ps with high confidence, owing to our obtainable high signal-to-noise ratio (SNR) and strongly Gaussian instrument response function (IRF; Fig. S4b);^41^ we also note that it is easier to resolve a delayed rise-time than it is to quantify a lifetime (analogous to determining a peak position rather than quantifying a lineshape). This explanation would also fail to explain why a peak is observed at *ω*_IR_ = 2690 cm^−1^ in 2D-BonFIRE, which is well below any reasonably expected CH-stretching frequency.

Instead, we suspect that the IR transitions we excite in the CH-stretching region are not fundamental modes but are actually combination modes involving the simultaneous excitation of two fundamental modes, but only one of which is strongly FC-coupled. In this case, one would expect that only the FC-coupled mode would contribute to double-resonance, which would lead to a violation of the resonance condition. These assignments agree with the prompt (non-delayed) rise-times (Fig. S5a) and are additionally validated by anharmonic DFT calculations (Fig. S5b), which predict several strong IR-active combination modes in the CH-stretching region for Rh800. This interpretation is supported by further control experiments and analysis (Section S4), including the absence of CD-stretching BonFIRE in a deuterated dye (Fig. S6), experimental confirmation that our temporal resolution is sufficient to observe delayed rise-times (Fig. S7), and the time-evolution of the *ω*_probe_-dependence in 2D-BonFIRE (Fig. S8). We compare several possible signal mechanisms in Fig. S9, showing that only directly excited combination modes are fully consistent with our experimental data.^67^

We further tested additional dyes, including ICG, ICG-d_7_, ATTO665, ATTO680, ATTO725, and Cy5.5, consistently observing similar violations of the resonance condition in the 2600–3200 cm^−1^ region (Fig. S10). We summarize all our results in Fig. 2f by plotting the redshift from the UV-vis profile for each measured mode (quantified as *ω*_max_ − *ω*^peak^_probe_) *versus* the IR-excitation frequency for all measured molecules (also see Table S2). Visibly, violations of the resonance condition are consistently observed in the rhodamine-based dyes measured here. Interestingly, strong combination modes in the CH-stretching region have been reported to be a hallmark of the rhodamine scaffold (and perhaps aromatic molecules in general),^68–70^ and these modes are consistently visible in rhodamine-based dyes but absent in cyanine-based dyes in our experiments (Fig. S10). The clear structural dependence of these features may also help to explain why our dyes exhibit characteristically different spectra than the coumarins studied by Kaiser and co-workers and the azaindoles studied by Sakai. Furthermore, we note that our assignments of the combination modes of Rh800 (Table S1) agree well with previous combination mode assignments for rhodamine 6G by Majoube and Henry, which implies that our experimental observations and interpretation here are likely more general for rhodamine-based dyes.^70^

Ultimately, further experiments and theory are necessary to prove that the observed features are combination modes. For example, it would be valuable to synthesize and characterize a fully deuterated analogue of Rh800, which is theoretically feasible but not trivial, since all the synthetic precursors would also need to be prepared from simple deuterated building blocks.^28,71^ In future work, we also plan to explore the quantum mechanical basis for these observations. Regardless, compared with the limited means to assign combination modes in existing 1DVF methods (either by kinetic modeling^31^ or by the observed IR frequency alone^65,72,73^), the higher dimensionality of 2D-BonFIRE indeed provides an improved framework for identifying combination modes.

### 2D-BonFIRE reveals heterogeneity in vibrational cooling

**Fig. 3:**
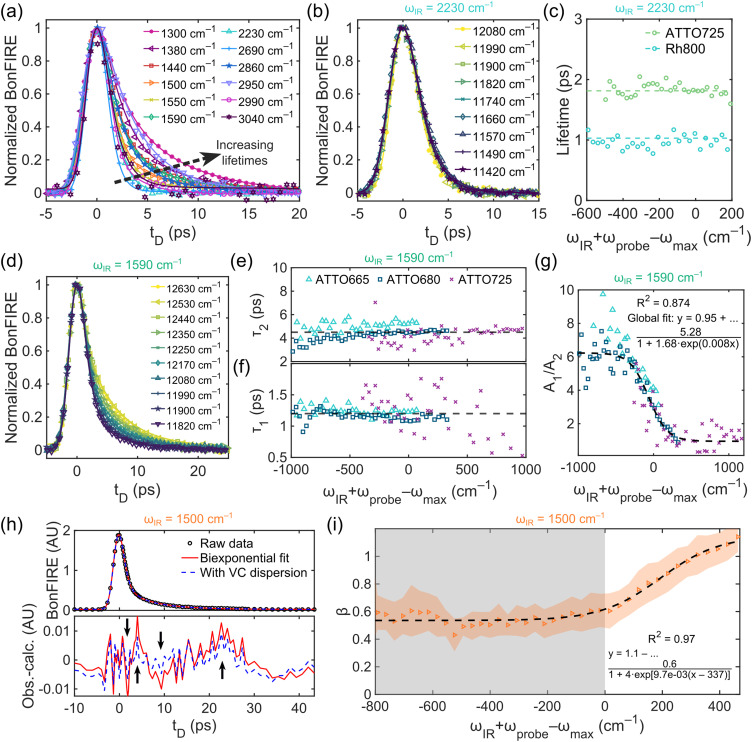
2D-BonFIRE time-domain spectroscopy. (a) Representative normalized time-domain spectra of major modes of Rh800. (b) Normalized time-domain spectra of the nitrile-stretch of ATTO725 at *ω*_IR_ = 2230 cm^−1^ and varying *ω*_probe_. (c) *ω*_probe_-dependence of the nitrile lifetimes of Rh800 (blue) and ATTO725 (green) at *ω*_IR_ = 2230 cm^−1^. The nitrile lifetimes are constant, characteristic of a local oscillator mode. (d) Normalized time-domain spectra of ATTO725 at *ω*_IR_ = 1590 cm^−1^ and varying *ω*_probe_. (e–g) *ω*_probe_-dependence of (e) *τ*_2_ (dashed line at 4.5 ps), (f) *τ*_1_ (dashed line at 1.2 ps), and (g) *A*_1_/*A*_2_ (trendline is a sigmoidal global fit) for ATTO665, ATTO680, and ATTO725 at *ω*_IR_ = 1590 cm^−1^. (h) Rh800 time-domain BonFIRE at *ω*_IR_ = 1500 cm^−1^ and *ω*_probe_ = 12 950 cm^−1^. Judging by the oscillating residuals of the fit (obs.–calc.; bottom panel), the observed decay is not described by a pure biexponential (red solid line; residual oscillations highlighted by black arrows). Modelling VC rate dispersion with a KWW function (blue dashed line) yields improved fitting. (i) *β* as a function of probing energy for *ω*_IR_ = 1500 cm^−1^ (trendline is a sigmoidal fit). *β* transitions from its initial value of ∼0.5 to ∼1 as *ω*_probe_ increases.

Going beyond the frequency domain, we then explored how 2D-BonFIRE changes as a function of the time delay (*t*_D_) between the IR and probe pulses. These measurements should inform the decay of the excited vibrational population on the picosecond timescale, allowing for previously inaccessible direct tracking of vibrational dynamics with ultrasensitive fluorescence detection.^41^ Deconvolution with our 2.5 ps Gaussian IRF (measured by NDR-TPA; Fig. S4b) and our high SNR allow for highly accurate fitting of even subpicosecond dynamics, as we and others have shown previously.^41,61,74^ Furthermore, thanks to our narrowband (10 cm^−1^) pulses for mode-selective excitation, BonFIRE excels in observing the dynamics of individual vibrational modes without the confounding effects of multimode coherences.^35,36^Fig. 3a shows representative time-domain spectra of a series of vibrational modes of Rh800, highlighting the diverse dynamics that exist within a single molecular structure. For our analyses, we assume that we excite only one vibrational mode within the bandwidth of our IR pulse, which is well-supported by DFT calculations^41^ and the fact that we do not observe oscillations from multimode coherences.^23^

In the time domain, we first examined 2D-BonFIRE of the nitrile-stretching vibration (Fig. 3b). As a “local oscillator”, the nitrile resonates in a frequency region with a low density of states (DOS), and thus it presents as an ideal model vibration, decaying with a single lifetime (*τ*) due to intramolecular vibrational-energy redistribution (IVR).^41^ Generally, local oscillators like nitriles, carbonyls, and alkynes feature monoexponential decays (exp(−*t*_D_/*τ*)), though there can be exceptions, depending on a molecule's local mode structure.^36^

To compare lifetimes as a function of excitation energy, we introduce *ω*_IR_ + *ω*_probe_ − *ω*_max_ as the *x*-axis in Fig. 3c. The nitrile vibrational lifetimes of both ATTO725 and Rh800 are observed to be independent of *ω*_probe_, which is expected in the ideal case because the lifetime of the vibrational population should be a property of the excited mode and not be influenced by *ω*_probe_ (*i.e.*, *ω*_IR_ should determine the observed dynamics; the pulse widths of both pulses affect the raw time-domain signals, but this is accounted for with deconvolution).^41^

Interestingly, quite a different case presents for the other modes (Fig. 3d). Unlike the nitrile-stretching modes, the ring-breathing modes (and their combination modes) exist in high-DOS frequency regions, leading to overall biexponential decays ((*A*_1_/*A*_2_)exp(−*t*_D_/*τ*_1_) + exp(−*t*_D_/*τ*_2_)).^13,32,61,62^ The fast decay (*τ*_1_) is attributed to IVR among local states,^63^ and the slow decay (*τ*_2_) is attributed to subsequent vibrational cooling (VC)^75^*via* energy transfer to the solvent.^76,77^ In large molecules, the DOS is high enough to be treated as a continuum (*e.g.*, Rh800 has 162 normal modes),^41^ meaning that, rather than thinking about the populations of individual modes, we consider vibrational relaxation from the perspective of the vibrational distribution across all of the modes (which tends towards a Boltzmann distribution in shape).^78^ Following IVR, the vibrational energy distribution is described as a ‘hot’ Boltzmann distribution (since IR energy was added to the system), and through VC, the distribution ‘cools’ towards a room-temperature distribution (Section S5).^54^

Furthermore, the fingerprint decays appear elongated as *ω*_probe_ increases (Fig. 3d). As a model fingerprint mode, we examine the 1590 cm^−1^ ring-breathing mode of ATTO665, ATTO680, and ATTO725. Surprisingly, we found that as the probing energy increases, both *τ*_1_ and *τ*_2_ remain constant (1.2 ps and 4.5 ps, respectively; Fig. 3e and f). The observed decay elongation is entirely due to *A*_1_/*A*_2_ decreasing sigmoidally from ∼6 to ∼1 (Fig. 3g).^63^ The decrease of *A*_1_/*A*_2_ indicates that 2D-BonFIRE becomes increasingly immune to IVR as *ω*_probe_ increases, since up-conversion becomes possible from decayed vibrational states (Fig. S2; see also Section S5 and Fig. S11). Additionally, the inflection point of the fit is within error of *ω*_IR_ + *ω*_probe_ = *ω*_max_, indicating that the resonance condition marks the midpoint of the sigmoid. This behaviour is quite interesting, given that from a simple kinetic picture, one might expect that *τ*_1_ and *τ*_2_ should directly influence *A*_1_/*A*_2_. However, in 2D-BonFIRE, *A*_1_/*A*_2_ reflects the sensitivity of the up-conversion to IVR relative to VC (*i.e.*, the relative *f*_FC_ of the pre-IVR and post-IVR vibrational distributions). Thus, in the ideal case, *τ*_1_ and *τ*_2_ are independent of *A*_1_/*A*_2_ and governed solely by the S_0_ vibrational potential. These data illustrate 2D-BonFIRE's potential as a direct, experimental means of mapping local *f*_FC_ distributions and establish the *A*_1_/*A*_2_ ratio as a meaningful observable in vibronic fluorescence spectroscopies.

Interestingly, when we examine the 1500 cm^−1^ ring mode of Rh800, we observe deviations from ideal biexponential character, as is evident in the residuals of the fit (red solid curve, Fig. 3h; deviations highlighted by black arrows). These oscillations are small (∼1% of the signal size), but we are confident that such deviations are not baseline noise due to the high SNR (∼430 in Fig. 3h) in our measurements. These features are also present in the two other strong fingerprint modes of Rh800 (1300 cm^−1^ and 1590 cm^−1^) and develop on different timescales for the different modes (Section S6 and Fig. S12), indicating the deviations are a molecular response rather than an instrumental artifact.

To understand these deviations, we consider that an exponential decay describes a process that occurs with a fixed lifetime. Picturing a homogeneous ensemble of oscillators, one would expect each oscillator to decay with the same rate; if we plotted the distribution of rates across a homogeneous ensemble, the result would be a delta function (all oscillators decay at the same rate). However, if there is heterogeneity (*e.g.*, some oscillators decay faster or slower compared to others), then the distribution of rates takes on a nonzero width: such a case is termed “rate dispersion” and presents as deviation from ideal exponential character.^79^ The mechanistic origins of rate dispersion can be further classified as heterogeneous rate dispersion (*e.g.*, different molecules experience different local environments) and homogeneous rate dispersion (*e.g.*, each molecule reports a non-exponential decay due to the presence of multiple relaxation pathways).^79^

It has long been thought that VC inherently exhibits rate dispersion.^75^ In large molecules, there are many possible VC pathways, due to the large DOS, and each possible pathway can have a different rate (see Fig. S12d for a conceptual illustration). Therefore, rate dispersion in VC can manifest even in a homogeneous sample, as the dispersion results from the different possible VC pathways of an individual molecule (*i.e.*, homogeneous rate dispersion). Given that we may probe multiple possible VC pathways across the ensemble, it follows that our expected VC lineshape becomes a continuous sum of exponential decays. Such lineshapes can be described by the Kohlrausch–Williams–Watts (KWW) function (exp[−*t*_D_/*τ*_2_]^*β*^),^80^ where *β* represents the degree of sampled dispersion (further from 1 indicates more dispersion).^81,82^

Upon refitting our data, replacing only the slow decay in the biexponential fit with a KWW function, the fits converge visibly better (blue dashed curve, Fig. 3h). When we examine the dependence of *β* on *ω*_probe_, we observe a striking trend: *β* transitions in sigmoidal fashion (*R*^2^ = 0.97) from ∼0.5 to ∼1 (within experimental error) as *ω*_probe_ increases (Fig. 3i). This clear trend is only visible in our highest-SNR 2D-BonFIRE data (*ω*_IR_ = 1500 cm^−1^), since the SNR requirements are quite stringent for accurate fitting of a biexponential with VC rate dispersion (trends in *ω*_IR_ = 1300 and 1590 cm^−1^ are shown in Fig. S12c). Importantly, the dependence of *β* on *ω*_probe_ indicates the involvement of the dye's vibronic structure, in agreement with a homogeneous rate dispersion mechanism.

For *ω*_IR_ = 1500 cm^−1^, our data suggest that the observed VC decay is dispersed at low *ω*_probe_, but it becomes less dispersed as *ω*_probe_ increases. One possible interpretation of this trend stems from the picture of multiple VC pathways, where different pathways involve loss of different amounts of energy to the solvent (see Fig. S13 for a conceptual illustration). In the low-*ω*_probe_ limit, even a small loss of energy can mean that a molecule no longer has enough energy to be up-converted to 
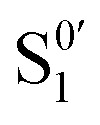
 (Fig. S13b). In this case, BonFIRE is sensitive to many possible VC pathways, giving rise to VC dispersion. However, as *ω*_probe_ increases and decayed vibrational states can be up-converted (Fig. S13c), BonFIRE loses sensitivity to the low-energy-loss VC pathways, becoming increasingly selective for only the VC pathways that result in larger energy losses (and thereby becoming less dispersed).

To our knowledge, this is the first direct observation of homogeneous VC rate dispersion, which perhaps is not surprising given the strict requirements of high SNR and broad tunability necessary to resolve such minute features in an experimental measurement. Significant theoretical work is needed to better understand these observations. This measurement also demonstrates that 2D-BonFIRE serves as a complement to existing nonlinear vibrational spectroscopies like 2DIR and other methods,^83,84^ since this VC dispersion is observed by probing much of the DOS (where most of the modes may not be strongly IR-active but can still be FC-active and up-converted in 2D-BonFIRE). Such information would also not be easily obtained by 2D electronic methods (which can report vibronic information in the S_1_ manifold), as we selectively probe vibrational dynamics within the S_0_ manifold in 2D-BonFIRE. Thus, the unique capabilities of 2D-BonFIRE allow for new mechanistic insights into vibrational relaxation. In future work, it would be highly informative to integrate 2D-BonFIRE with other 2D techniques, such as 2DVE, to better understand these complex dynamics.

### 16-Colour imaging with 2D-BonFIRE microscopy

We reasoned that the dependence of 2D-BonFIRE on both the vibrational and electronic structure of a molecule should allow 2D super-multiplex imaging with a significantly enlarged pool of “vibronic colours”. While conventional fluorescence imaging is limited to 4–6 colours due to inherently broad spectra,^71^ vibrational imaging approaches have demonstrated up to 14 vibrational colours in a single shot,^28,85,86^ eliminating the need for multiple rounds of labeling.^87,88^ Compared to purely vibrational imaging approaches (*ω*_IR_ only), 2D-BonFIRE can explore the dimensions of both *ω*_IR_ and *ω*_probe_, offering a new metric for increasing multiplexing while achieving single-molecule sensitivity with our current optimizations (Section S7, Fig. S14 and S15).

**Fig. 4:**
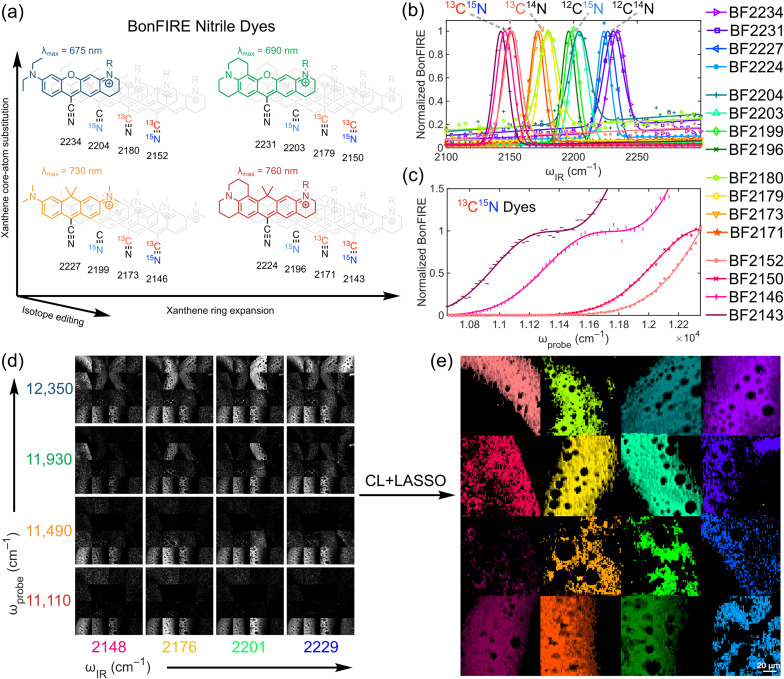
16-colour chemical imaging by 2D-BonFIRE. (a) Structures of BonFIRE nitrile dyes for super-multiplex imaging (R = (CH_2_)_3_CO_2_^−^). Dye scaffolds are colour-coded by electronic absorption and labelled with their absorption maxima,^71^ and individual dyes are labelled by their nitrile IR frequency in cm^−1^. (b) Lifetime-weighted IR reference spectra for BF dyes. (c) Probe reference spectra for ^13^C^15^N BF dyes (other isotopologues shown in Fig. S16). (d) Stitched BonFIRE images of labelled PS films acquired with varying *ω*_IR_ and *ω*_probe_ (contrast scaled for each *ω*_probe_ for visual comparison). (e) Unmixed 16-colour BonFIRE image of labelled PS films by adding conditional logic to the least absolute shrinkage and selection operator (CL + LASSO). Unmixed component images are provided for reference in Fig. S18.

To evaluate 2D-BonFIRE's multiplexing capabilities, we synthesized a set of 16 nitrile dyes,^28,71^ distinguished by the nitrile isotopologue and the electronic scaffold to which the nitrile is attached (Fig. 4a). It is well-established that the four main nitrile isotopologues (^12^C≡^14^N, ^12^C≡^15^N, ^13^C≡^14^N, and ^13^C≡^15^N) are vibrationally distinct, while the isotopic substitutions have no discernible effect on a dye's electronic absorption spectrum.^38^ Thus, nitrile isotopologues and fluorescent scaffolds can be ‘mixed and matched’ to yield a vibronically diverse palette (Fig. 4a), where the nitrile isotopologues are well-separated in *ω*_IR_ (Fig. 4b) and the different electronic scaffolds are well-separated in *ω*_probe_ (Fig. 4c). For convenience, we have named each dye by its peak IR frequency in BonFIRE (Fig. 4a).

To demonstrate 2D-BonFIRE's imaging capability, we drop-cast 16 polystyrene (PS) films onto a single window, each individually labelled with a different dye, and acquired BonFIRE images of each of the films at each combination of *ω*_IR_ = 2229, 2201, 2176, and 2148 cm^−1^ and *ω*_probe_ = 12 350, 11 930, 11 490, and 11 110 cm^−1^ (Section S8 and Fig. S16) for a total of 16 BonFIRE images. The hyperspectral images of the 16 films were stitched into a single hyperstack (Fig. 4d), allowing us to directly benchmark our ability to unmix spectrally overlapping dyes in 2D-BonFIRE. By adding conditional logic to the least absolute shrinkage and selection operator (CL + LASSO; Fig. S17),^89,90^ we achieved robust unmixing of our 16 dyes with minimal crosstalk (Fig. 4e and S18). To our knowledge, this demonstration features the largest number of resolved vibrational colours in a far-field imaging modality.^91^

It is important to note that with a traditional 1D method (either electronic or vibrational), only four colours would be resolvable from this set of 16 dyes. 2D-BonFIRE, with 2D spectral information, is uniquely capable of resolving all 16 colours. Furthermore, we note that we achieved 16-colour imaging with only near-IR nitrile dyes (*λ*_max_ from 675–760 nm). By incorporating the fingerprint region and additional probe frequencies (15 000–25 000 cm^−1^), we aim to eventually achieve ultra-multiplex BonFIRE imaging (>25 colours).

**Fig. 5:**
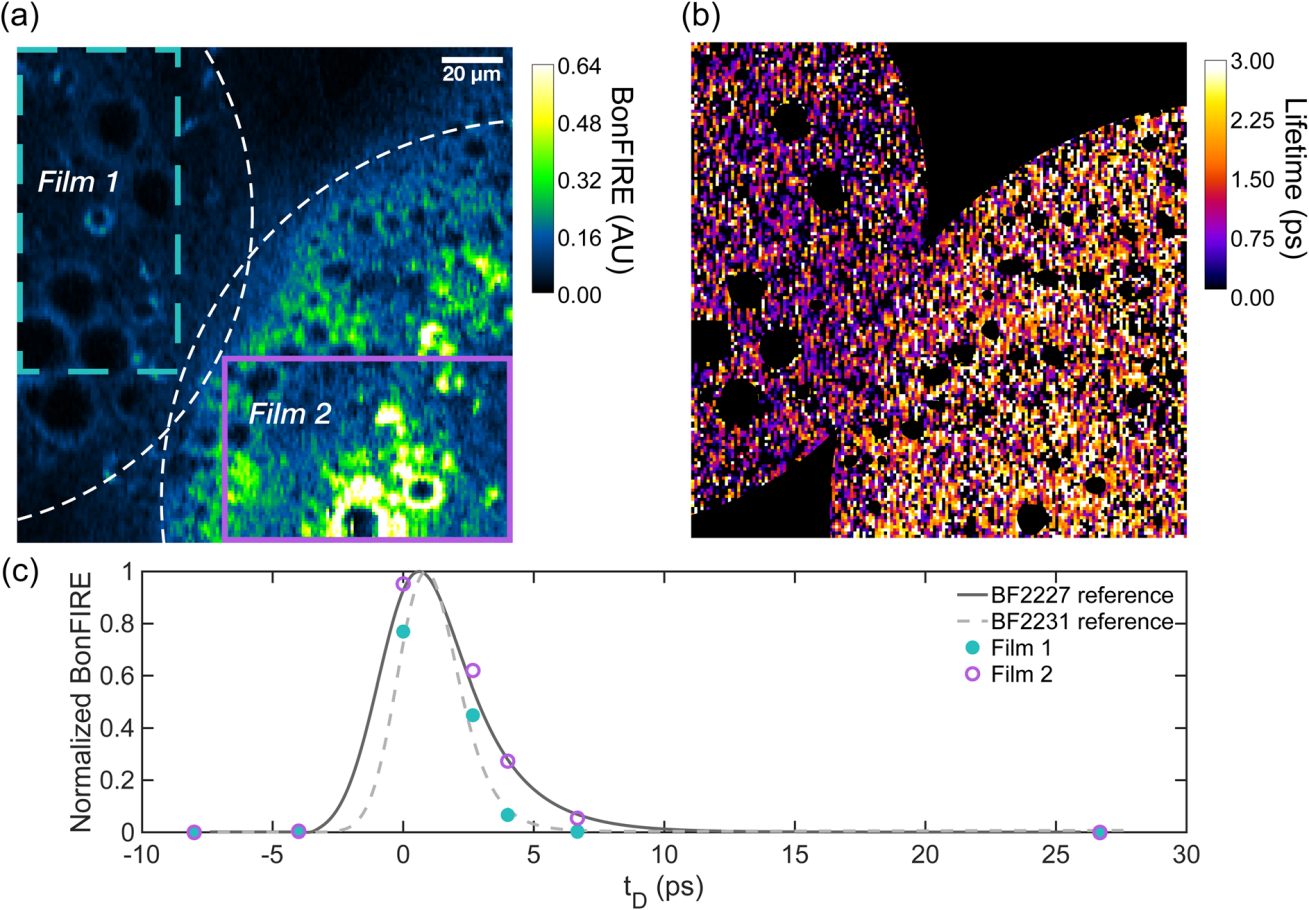
Vibrational lifetime multiplex imaging. (a) BonFIRE image of overlapping BF2227 and BF2231 films at *ω*_IR_ = 2229 cm^−1^ and *ω*_probe_ = 11 930 cm^−1^ (white dashed lines mark the approximate edges of the films). (b) Vibrational lifetime image of overlapping BF2227 and BF2231 films (low-intensity regions are masked). Film 2 exhibits systematically larger vibrational lifetimes. (c) Overlay of reference temporal profiles of BF2227 (dark grey solid curve) and BF2231 (light grey dashed curve) and temporal profiles of films 1 (turquoise dots) and 2 (purple circles) in the regions of interest marked in panel (a). The temporal profiles of films 1 and 2 align with the solution spectra of BF2231 and BF2227, respectively.

Super-multiplex imaging can be achieved by other imaging modalities, including pre-resonance stimulated Raman scattering (SRS) microscopy (though with slightly lower demonstrated multiplex imaging than 2D-BonFIRE).^28,92^ However, a capability unique to BonFIRE is yet a third dimension, vibrational lifetime multiplex imaging, where two dyes overlapping vibrationally and electronically could be differentiated by their characteristic vibrational decays. To demonstrate this novel concept, we imaged the interface of our BF2227 and BF2231 PS films (Fig. 5). At *ω*_IR_ = 2229 cm^−1^ and *ω*_probe_ = 11 930 cm^−1^, both films exhibit appreciable BonFIRE (Fig. 5a). However, their vibrational lifetimes are *τ*_BF2227_ = 1.9 ± 0.2 ps and *τ*_BF2231_ = 1.1 ± 0.1 ps, which are clearly separable in the time domain. Therefore, even with sparse sampling (Section S9 and Fig. S19), the two films can be readily differentiated based on their vibrational lifetimes (Fig. 5b). When overlaid with the solution reference measurements, the imaging results align quite well (Fig. 5c), allowing us to confidently identify Film 1 as containing BF2231 and film 2 as containing BF2227.

## Conclusion and outlook

In this work, we have demonstrated 2D-BonFIRE spectroscopy and imaging. In the frequency domain, 2D-BonFIRE allowed for direct observation of violations of the resonance condition, providing strong experimental evidence of combination modes in the CH-stretching region. Beyond fundamental motivations, combination modes are currently gaining interest toward understanding the vibrational dynamics that drive chemical reactions.^93^ Given the single-molecule sensitivity of BonFIRE,^38–40^ 2D-BonFIRE could allow such studies to be extended to single-molecule reaction conditions, allowing for new insights into chemical reactivity.^13,17^ To our knowledge, 2D-BonFIRE is the first 2D vibrational spectroscopy with single-molecule sensitivity. (Early work from Mastron and Tokmakoff demonstrated a fluorescence-detected analogue of 2DIR, but they noted that its sensitivity was limited due to their two-photon up-conversion to generate fluorescence.)^24^

In the time domain, 2D-BonFIRE revealed mode-specific dynamics and VC rate dispersion, illuminating the inherent heterogeneity of vibrational relaxation. Given that vibrational lifetimes can act as local sensors,^41,62^ 2D-BonFIRE could be an invaluable tool for characterizing photophysical properties of proteins with fluorescent chromophores, such as the newly discovered rhodopsin-cyclases (*e.g.*, neorhodopsin, a bright, near-IR fluorescent protein).^94^ Current literature calls for deeper spectroscopic and structural investigations toward understanding the dynamics of these proteins.^95^ The state-of-the-art method for studying these systems is resonance femtosecond SRS, which uses the resonance-enhancement of the chromophore to reject Raman background from the rest of the protein.^96^ In a similar manner, 2D-BonFIRE can selectively probe these chromophores in their intrinsic environments and reject protein background, but theoretically with single-molecule sensitivity. An especially interesting experiment would entail using 2D-BonFIRE as a probe following a separate optical excitation (*i.e.*, transient 2D-BonFIRE), observing photoexcited vibrational dynamics through the evolution of 2D-BonFIRE over time. These works may be facilitated by incorporating lifetime distribution analysis techniques, such as the maximum entropy method or an inverse Laplace transform.^97,98^

Finally, we showed proof-of-concept 16-colour super-multiplex imaging (to our knowledge, setting the multiplexing record for far-field chemical imaging) and vibrational lifetime multiplex imaging with 2D-BonFIRE. With our demonstrated imaging capabilities, applications like optical barcoding with PS beads should be readily achieved.^87^ However, a major shortcoming of our current implementation is imaging speed. If we instead adopt a wide-field imaging configuration^40^ and spatially overlap multiple lasers of differing frequencies (but displace them temporally to still allow for independent signal acquisition), such “multicolour BonFIRE” could be orders of magnitude faster than our current implementation, facilitating live-cell applications where speed is essential. Faster imaging could also allow for finer sampling in *t*_D_ and frame averaging, granting higher SNR and improved lifetime imaging performance. With the single-molecule sensitivity of BonFIRE,^39^ achieving super-multiplexing with 2D-BonFIRE represents a significant step toward super-multiplex vibrational single-molecule localization microscopy.

## Author contributions

Conceptualization and writing – original draft: P. A. K. and L. W. (equal). Methodology, formal analysis, and investigation: P. A. K. (lead); all authors (supporting). Validation and writing – review & editing: all authors. Funding acquisition, project administration, resources, and supervision: L. W.

## Conflicts of interest

There are no conflicts to declare.

## Supplementary Material

SC-016-D5SC02628H-s001

## Data Availability

Data for this article are available on Figshare (https://doi.org/10.6084/m9.figshare.28655882). Methods, additional discussions (2D-BonFIRE in the high-probe energy limit, non-degenerate resonance-enhanced two-photon absorption, additional evidence of combination modes in 2D-BonFIRE, vibrational relaxation in 2D-BonFIRE, vibrational cooling rate dispersion in 2D-BonFIRE, single-molecule sensitivity of 2D-BonFIRE, unmixing of hyperspectral 2D-BonFIRE images by CL + LASSO, and vibrational lifetime imaging with sparse sampling), supplementary tables, and references are provided in the Supplementary information. See DOI: https://doi.org/10.1039/d5sc02628h.
