# Supplementary material for: Two-dimensional bond-selective fluorescence spectroscopy: violations of the resonance condition, vibrational cooling rate dispersion, and super-multiplex imaging
Source: Chem Sci. 2025 Jul 30;16(33):14905–18. doi: 10.1039/d5sc02628h (PMC12336853; doi:10.1039/d5sc02628h)
Supplement: SC-016-D5SC02628H-s001 [file SC-016-D5SC02628H-s001.pdf]

Supplementary Information (SI) for Chemical Science.
This journal is © The Royal Society of Chemistry 2025

Supplementary Information

Two-dimensional bond-selective fluorescence spectroscopy: violations of the resonance condition, vibrational cooling rate dispersion, and super-multiplex imaging

Philip A. Kocheril, Jiajun Du, Haomin Wang, Ryan E. Leighton, Dongkwan Lee, Ziguang Yang, Noor Naji, Adrian Colazo, and Lu Wei*

Division of Chemistry and Chemical Engineering, California Institute of Technology, Pasadena, CA 91125, USA

*Corresponding author: lwei@caltech.edu

Table of Contents

S1. Methods. ***S3***

2D-BonFIRE instrumentation. S3

Figure S1. Simplified instrument diagram for 2D-BonFIRE. S3

Dyes. S5

Solution sample preparation. S6

Solution spectra acquisition. S6

Polymer film preparation. S7

Polymer film imaging. S7

Data analysis and visualization. S8

Computational methods. S9

Steady-state spectral characterizations. S10

S2. 2D-BonFIRE in the high-probe energy limit ***S11***

Figure S2. BonFIRE energy-level diagrams as a function of probing energy. S11

Figure S3. 2D-BonFIRE spectra of other dyes. S13

S3. Non-degenerate resonance-enhanced two-photon absorption. ***S15***

Figure S4. Non-degenerate resonance-enhanced two-photon absorption. S16

S4. Additional evidence of combination modes in 2D-BonFIRE. ***S19***

Figure S5. BonFIRE rise-time in combination modes and anharmonic DFT. S19

Figure S6. Absence of CD-stretching BonFIRE in ICG-d_7. .... S20

Figure S7. Delayed rise-time at 1100 cm^{-1}. S21

Figure S8. Time-evolution of probe-dependence in 2D-BonFIRE. S23

Figure S9. Comparison of BonFIRE signal mechanisms. S24

Figure S10. Combination modes in 2D-BonFIRE with other dyes. S25

S5. Vibrational relaxation in 2D-BonFIRE. ***S26***

Figure S11. Mechanistic picture of vibrational relaxation. S27

S6. Vibrational cooling rate dispersion in 2D-BonFIRE. ***S29***

Figure S12. Vibrational cooling rate dispersion in other vibrational modes. S30

Figure S13. Conceptual illustration of VC rate dispersion in the 1500 cm^{-1} mode of Rh800. .... S32

S7. Single-molecule sensitivity of 2D-BonFIRE. ***S33***

Figure S14. 2D-BonFIRE in highly dilute solutions of Rh800. .... S33

Figure S15. Saturated mid-IR absorption in 2D-BonFIRE. S34

S8. Unmixing of hyperspectral 2D-BonFIRE images by CL+LASSO...S35
Figure S16. Probe reference spectra and unfolded hyperspectra of individual BonFIRE nitrile dyes. S36
Figure S17. CL+LASSO implementation. S37
Figure S18. Unmixed 16-color component images from CL+LASSO. S38

S9. Vibrational lifetime imaging with sparse sampling. S39
Figure S19. Non-convolution lifetime fitting. S40

Supplementary Tables. S41

Table S1. Summary of 2D-BonFIRE data on Rh800. ....S41

Table S2. Summary of 2D-BonFIRE frequency-domain data across all molecules. S42

References...S43

S1. Methods.

2D-BonFIRE instrumentation. The instrumentation for 2D-BonFIRE is a modified version of our previously reported BonFIRE microscope (**Fig. S1**).[1] A 1.6-ps, 10-cm^{-1}, 80-MHz, mode-locked Yb fiber laser (aeroPULSE PS10, NKT Photonics; customized high-power version) is used to pump two optical parametric oscillators (OPOs). The first OPO (Levante IR, Applied Physics and Electronics; customized tuning range) uses the 1031.2-nm pump directly, generating signal (1320-2000 nm) and idler (2150-4800 nm) beams. To study vibrations in the cell-silent and CH-stretching regions, the mid-IR idler is focused into and collimated out of an acousto-optic modulator (AOM; GEM-40-4-4500/4 mm, Brimrose) with a pair of lenses (LA5255-E, Thorlabs). The AOM is driven by a 1.05 MHz (chosen to avoid noise from an environmental harmonic at 1 MHz), 5 Vpp square wave from an arbitrary waveform generator (AWG; DG2102, RIGOL) with a 35% duty cycle.[2]

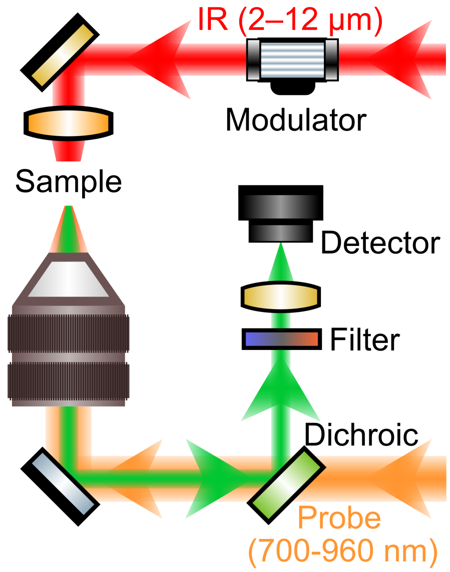

Figure S1. Simplified instrument diagram for 2D-BonFIRE.

To study vibrations in the fingerprint region, the Levante signal and idler beams are used for difference-frequency generation (DFG; HarmoniXX DFG, Applied Physics and Electronics;

5-12 μm). Phase-matching is optimized by maximizing the DFG power on a thermopile sensor (919P-003-10, Newport) connected to a power meter (843-R, Newport). The DFG pulse train is modulated by a chopper at 10 kHz (MC2000B, Thorlabs) or by an AOM (GEM-40-4-6282/2 mm, Brimrose) driven at 1.05 MHz, again focused and collimated with a pair of lenses (LA7228-E2, Thorlabs).

The mid-IR beam (idler or DFG) is then expanded by a pair of off-axis parabolic mirrors (37-242 and 37-243, Edmund Optics) prior to being routed to a ZnSe lens (39-469, Edmund Optics) with ~60% transmission efficiency at the top of the microscope (CEA1600, Thorlabs). The chopper for the DFG pulse train is placed at the focus of the parabolic mirror telescope. High-reflectivity gold mirrors (PF10-03-M01 for the idler, PF10-03-M02 for the DFG; Thorlabs) are used throughout the optical path. The DFG optical path (up to the ZnSe lens) is purged with N_2 using a custom purging enclosure comprising an epoxy-sealed acrylic box, lens tubes (SM1L series, Thorlabs), and plastic sheaths. Purging efficacy is monitored with a hair hygrometer (2131K5, McMaster-Carr), which consistently read < 1% relative humidity during measurements.

In the second OPO (picoEmerald S, Applied Physics and Electronics), the 1031.2-nm pump is frequency-doubled prior to parametric down-conversion, generating a signal beam (700-960 nm) in the visible–to–near-IR and an idler beam (1080-1950 nm) in the near-IR. The signal beam ("probe") is separated from the idler with a 1000-nm dichroic mirror (DMLP1000, Thorlabs), reflected in two separate passes off a silver retroreflector on a delay stage (DL-BKIT2U-S-M and DL325, Newport), and routed to a water-immersion 25× objective lens (XLPLN25XWMP2, Olympus) at the bottom of the microscope. Based on an internal power meter in the picoEmerald and using neutral density filters (NDK01, Thorlabs), probe power on-sample (after accounting for reflectivity losses and transmission losses from the objective) was about 1 mW for fingerprint-

region spectral measurements and imaging experiments, and 10 mW in the cell-silent and CH-stretching regions for solution spectral measurements. Broadband dielectric mirrors (BB1-E03, Thorlabs), silver mirrors (PF10-03-P01, Thorlabs), and ultrafast-enhanced silver mirrors (13-060, Edmund Optics) are used throughout the probe optical path. The polarizations of the IR and probe beams are kept parallel on the sample.

Fluorescence is captured through the water-immersion objective and separated from the signal beam by a dichroic beamsplitter (FF738-Di01 or FF801-Di02, Semrock) and bandpass filter (FF01-665/150 or FF01-709/167, Semrock). The reflected fluorescence is focused through a tube lens (AC254-200-B, Thorlabs) onto a photomultiplier tube (PMT; PMT1002, Thorlabs) with a 400-μm confocal pinhole (P400K, Thorlabs). The PMT gain was kept within its linear response range for all measurements (gain between 1 and 20), since nonlinearity (saturation behavior) occurs beyond 20. The output of the PMT (bandwidth 250 kHz for chopping, 2.5 MHz for AOM; DC offset +10 mV) is sent to a fast lock-in amplifier (210 MSa/s, 50 MHz; HF2LI, Zurich Instruments) and demodulated with a reference signal from either the chopper driver or the AWG (fourth-order time constant 3 ms). The resulting signal from the lock-in amplifier is sent through a terminal block (BNC-2110, National Instruments) to a data acquisition card (PCIe-6351, National Instruments) to be recorded in a custom LabVIEW virtual instrument (v19.0f2; National Instruments).

Dyes. Rhodamine 800 (Rh800; 83701, Millipore Sigma), ATTO665 (AD 665, ATTO-Tec), ATTO680 (AD 680, ATTO-Tec), ATTO725 (AD 725, ATTO-Tec), magnesium phthalocyanine (MgPc; 402737, Millipore Sigma), sulfo-cyanine5.5 (Cy5.5; A7330, Lumiprobe), indocyanine green (ICG; AMBH324A51A4, Millipore Sigma) and deuterated indocyanine green (ICG-d_7;

7749, Tocris Bioscience) were purchased commercially. BF nitrile dyes (**Fig. 4a**) were synthesized in-house using previously published syntheses.[3, 4] Dyes were stored as 10 mM stocks in dimethyl sulfoxide (DMSO; 276855, Millipore Sigma) or DMSO-d_6 (151874, Cambridge Isotope Laboratories) and kept at –20 °C until use.

Solution sample preparation. Except for MgPc, ICG, and ICG-d_7, dyes were diluted to 100 μM in DMSO-d_6 (for fingerprint and CH-stretching measurements) or DMSO (other measurements). ICG and ICG-d_7 were measured at 1 mM, and MgPc was measured in a saturated solution in DMSO (~290 μM).[5] After thoroughly mixing by pipetting, 1 μL of the desired solution was sandwiched between two CaF_2 windows (CAFP10-0.35 and CAFP25-0.5, Crystran) separated by a 6-μm Teflon spacer (MSP-006-M13, Harrick Scientific). The windows and spacer were cleaned with isopropanol (A451-4, Thermo Fisher Scientific) and lens tissue (MC-5, Thorlabs). The CaF_2 sandwich was placed in a sample holder comprising a lens tube (SM2L03, Thorlabs), a lens tube adapter (SM2A6, Thorlabs), and a thin metal plate with a 10-mm hole. The sample was secured against the metal plate with a 1” lens ring and mounted to a three-axis piezostage (P-545 PInano, Physik Instrumente) placed between the objective lenses.

Solution spectra acquisition. The ZnSe and water-immersion objective axial positions were adjusted with motorized mounts (ZFM2030 and ZFM2020, respectively; Thorlabs) controlled by a multi-axis motor controller (MCM3001, Thorlabs) to maximize the fluorescent signal intensity on the PMT. The pulses were laterally overlapped on the sample by using a multi-channel piezo controller (KIM101, Thorlabs) connected to two piezoelectric actuators (PIAK10, Thorlabs), maximizing signal by adjusting the x- and y-positions of the MIR objective relative to the NIR

objective. The time delay between the pulses was swept by adjusting the position of the delay stage, with a dwell time of 20 ms per acquisition and 25 replicate acquisitions per delay stage position (averaged together) for a given scan. The piezostage was used to acquire the replicates at different spatial positions on the sample.

Spectra were recorded by tuning ω_{IR} in 5- or 10-cm^{-1} steps and ω_{probe} in 2-nm steps. Acquiring the full fingerprint 2D spectrum of Rh800 confirmed that pumping on the high- or low-frequency side of a given IR resonance does not change the vibrational lifetime or probe spectrum, consistent with literature.[6] Thus, for all other samples, the IR frequency was swept to identify peaks, then the probe frequency was swept at each peak IR frequency.

Polymer film preparation. Polystyrene (PS; 430102, Millipore Sigma) was suspended in toluene (AA31755AP, Fisher Scientific) at 30 mg/mL and left to fully dissolve overnight. Each of the 16 dyes (from 10 mM stocks in DMSO) was individually diluted 1:100 into separate aliquots of the PS/toluene solution, making individual solutions at 100 μM. Older dye stocks that had taken on a significant amount of water were immiscible with toluene, which was remedied by adding 1 μL of dry DMSO as a cosolvent and slightly reducing the concentration. The dye/PS/toluene solutions were then drop-cast by loading 0.2 μL into a micropipette and dispensing as small of a volume as possible (~0.1 μL) onto a single 10-mm CaF_2 window, then allowing the toluene to evaporate. This process was performed for each of the 16 dye solutions, forming a 4×4 grid pattern on the window. The films were allowed to dry prior to imaging.

Polymer film imaging. Polymer films were imaged at each combination of: ω_{IR} = 2229, 2201, 2176, and 2148 cm^{-1}; ω_{probe} = 12,350, 11,930, 11,490, and 11,110 cm^{-1} (λ_{probe} = 810, 838, 870,

and 900 nm); and t_D = –8, 0, 2.7, and 26.7 ps. Images were acquired with a 0.5-ms time constant on the lock-in amplifier, a 1-ms pixel dwell time (fastest possible with our piezostage due to settling times), and a 1-µm step size in a raster-scanning modality, resulting in frame times of ~10 s. Laser tuning times ranged from 30 s to 2 min, leading to a total imaging time of ~40 min per film for all 16 frequency channels. All images were measured using the FF801-Di02 dichroic mirror and FF01-709/167 bandpass filter. For vibrational lifetime multiplex imaging, frames were acquired at t_D = –8, –4, 0, 2.7, 4, 6.7, and 26.7 ps (only at ω_{IR} = 2229 cm^{-1} and ω_{probe} = 11,930 cm^{-1}).

Data analysis and visualization. Frequency-domain data with multiple peaks were fit using Fityk (v1.3.1)[7] using Gaussian, Lorentzian, or Voigt lineshapes for peaks and a polynomial baseline as needed. When appropriate (i.e., not under saturation conditions), spectra were power-normalized. Time-domain data were sequentially fit as a function of t_D (positive t_D denotes that the NIR pulse arrives after the MIR pulse) using nonlinear least-squares fitting in MATLAB (lsqnonlin; R2024b, MathWorks).[2] Data with asymmetric t_D lists were interpolated as previously described.[2] Only data acquired with 1.05 MHz modulation (by AOM) were used for quantitative lifetime analysis. As a consequence of OPO tuning and dispersive optics (AOMs) in our optical paths, the arrival time of the picoEmerald signal changes with a rate of ~26 fs/nm (longer wavelengths arrive earlier), the arrival time of the Levante idler changes with a rate of ~6.9 fs/cm^{-1} (higher frequencies arrive later), and the arrival time of the DFG changes with a rate of ~2 fs/cm^{-1} (higher frequencies arrive later); these tuning rates necessitated correction of the time delay from absolute delay stage positions as a function of frequency, which was performed prior to fitting.

Data were fit to $I(t_D) = B(t_D) + IRF * \exp\left(-\frac{t_D}{\tau}\right)$ for monoexponential decays (nitriles) and $I(t_D) = B(t_D) + IRF * \left[\frac{A_1}{A_2}\exp\left(-\frac{t_D}{\tau_1}\right) + \exp\left(-\frac{t_D}{\tau_2}\right)\right]$ for biexponential decays ($*$ denotes convolution), using a Gaussian instrument response function (IRF).[2] Importantly, $\frac{A_1}{A_2}$ is used because the absolute values of A_1 and A_2 obtained from fitting are meaningless (the IRF also has a variable amplitude). The baseline (first 5% and last 20% of the data) was pre-fit to the sum of a line with a vertical offset and an exponential decay ($B(t_D)$). The pre-fit baseline coefficients were then passed to the convolution fit, allowing the coefficients to float ±10% of their pre-fit values. Data were fit by iteratively minimizing (towards zero) an error function defined as the experimental data subtracted from the fitting function. We employed strict convergence criteria in our fitting, allowing 10^6 function evaluations and fitting iterations. We additionally set our function tolerance, optimality tolerance, and step tolerance to 10^{-12}. Residuals of the fit were calculated by subtracting the fit from the experimental data.

Data were visualized in MATLAB, ImageJ (Fiji v2.14.0), and Inkscape (v1.3). Boltzmann population curves (**Fig. S11**) were generated by the product of the Boltzmann occupation factor ($\exp\left(-\frac{100hc\omega}{k_BT}\right)$) and the density of states (DOS)[8] (estimated as $\omega^{1.2}$ for Rh800) and normalized such that the area under each curve (representing the number of molecules present) is constant. For visualization of the 2D contour map shown in **Fig. 2a**, calculated BonFIRE intensities below the noise floor were replaced by the minimum measured intensity value ($5.47\cdot10^{-5}$ AU).

Computational methods. Molecular structure files were prepared in ChemDraw (v18.2; PerkinElmer, Waltham, MA, USA) and exported as 2D .mol file.[2] The .mol files were opened in Avogadro (v1.2)[9] to build a 3D geometry and for coarse optimization with the universal force field.

The optimized structure was exported from Avogadro as a .mol file and opened in GaussView (v6.1.1) to prepare the calculation job file.

DFT calculations were performed in Gaussian 16 (rev. B.01)[10] with the B3LYP functional and 6-31G(d,p) basis set. The molecular structure was subjected to fine geometry optimization, and ground-state vibrational frequency analysis was carried out. Raman analysis was disabled, and normal modes were explicitly saved. Vibrations were treated harmonically (anharmonic results came from previously reported calculations).[2] Natural bond orbital analysis was employed. A solvent cavity reaction field model for DMSO (ε = 47, unitless) was employed.

Steady-state spectral characterizations. For transmission FTIR measurements (Vertex 80v, Bruker), a 100 mM solution of Rh800 in DMSO (for the fingerprint and cell-silent regions) and a Rh800 pellet in KBr (for the CH-stretching region) were prepared.[1] Solutions at 1, 10, or 50 μM in DMSO were used for UV-vis absorption (Varian Cary 500, Agilent).[2]

S2. 2D-BonFIRE in the high-probe energy limit.

As described in the main text, we rationalize that BonFIRE in the region of $\omega_{probe} + \omega_{IR} > \omega_{max}$ is best considered in the framework of multi-state-to-multi-state transitions. To illustrate this point, we show BonFIRE energy-level diagrams for three distinct frequency regimes, differentiated by ω_{probe} (**Fig. S2**).

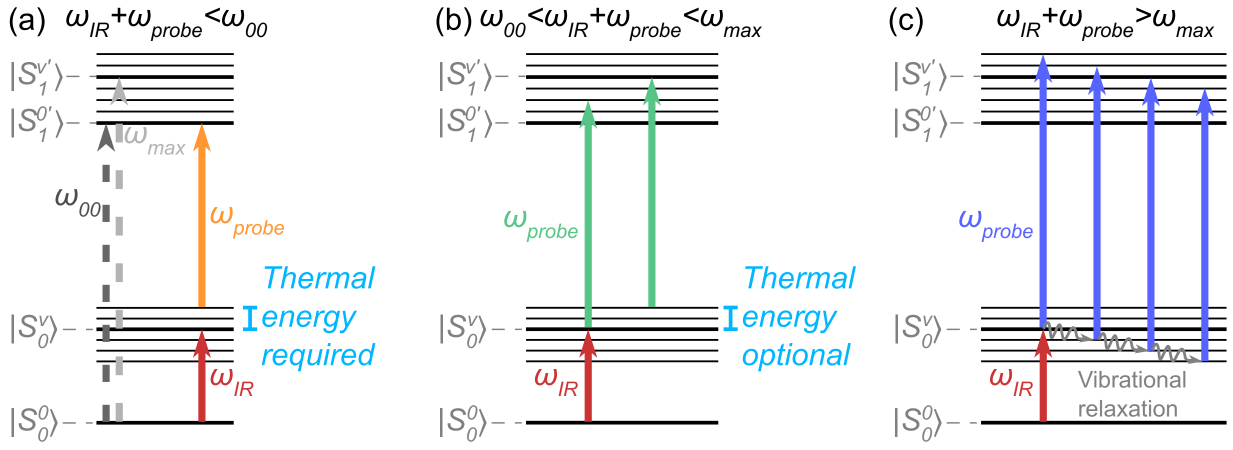

Figure S2. BonFIRE energy-level diagrams as a function of probing energy. (a) BonFIRE in the low-energy regime ($\omega_{probe} + \omega_{IR} < \omega_{00}$; dashed lines indicate ω_{00} and ω_{max}). Thermal energy is required to reach the S_1 manifold. (b) BonFIRE in the intermediate-energy regime ($\omega_{00} < \omega_{probe} + \omega_{IR} < \omega_{max}$). Thermal energy is no longer required to reach the S_1 manifold, but it is required to reach the absorption maximum at $|S_1^{v'}\rangle$. (c) BonFIRE in the high-energy regime ($\omega_{probe} + \omega_{IR} > \omega_{max}$). BonFIRE is possible from decayed vibrational states.

In the first regime, $\omega_{probe} + \omega_{IR} < \omega_{00}$ (**Fig. S2a**). As such, thermal energy is required for a molecule to be excited to the S_1 manifold, and the probed population in BonFIRE is small. Increasing ω_{probe} leads to more BonFIRE signal because the molecules require less thermal energy to be excited, and the dependence is roughly exponential as a consequence of the Boltzmann distribution. Eventually, an intermediate energy regime of $\omega_{00} < \omega_{probe} + \omega_{IR} <$

ω_{max} is reached (**Fig. S2b**). Here, ω_{probe} is of sufficient energy to excite molecules without any thermal energy, but BonFIRE signal continues to increase as the electronic absorption cross-section increases toward its maximum (as given by the resonance condition). Finally, in the high-energy regime of $\omega_{probe} + \omega_{IR} > \omega_{max}$, ω_{probe} is large enough that vibrationally relaxed molecules can still be excited to the S_1 manifold (**Fig. S2c**). In this regime, BonFIRE can be observed from a large set of vibrational states, and there are a correspondingly large set of potential states in the S_1 manifold to which molecules can be excited. As a result, the total BonFIRE signal continues to increase.

To test the generalizability of this multi-state-to-multi-state model, we measured 2D-BonFIRE spectra of six other popular, commercial dyes of varied structures: Cy5.5, ATTO665, ATTO680, ATTO725, MgPc, and ICG-d_7 (**Fig. S3**; structures of ATTO665 and ATTO725 are not publicly known). We observe quite similar trends to what we observed with Rh800 (**Fig. 2**), suggesting that this multi-state-to-multi-state framework is generalizable to large fluorescent molecules. We additionally note that the fingerprint spectra are unique (**Fig. S3a**, **f**), indicating that these dyes could be differentiated in a multiplexing context by their ω_{IR}-dependence.

The resonance condition is quite well-satisfied for Cy5.5 (**Fig. S3b**), ATTO665 (**Fig. S3c**) and ATTO680 (**Fig. S3d**). Interestingly, for ATTO725, we observe that the peak in the ω_{probe}-dependence in 2D-BonFIRE at ω_{IR} = 1590 cm^{-1} is blueshifted by ~250 cm^{-1} from its expected position (**Fig. S3e**), and a smaller blueshift of ~100 cm^{-1} is observed for the nitrile-stretch of ATTO725 (**Fig. S3e**).

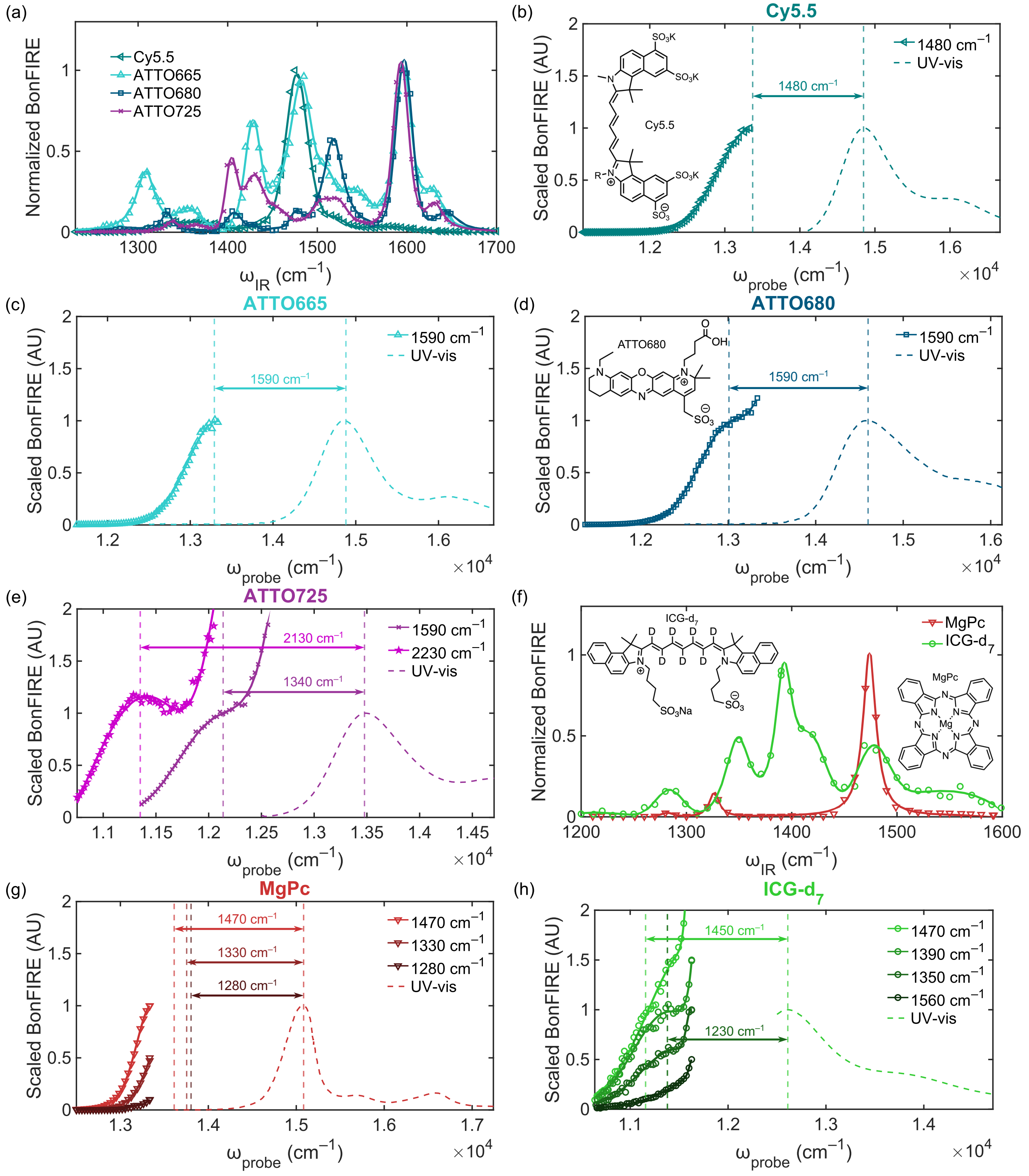

Figure S3. 2D-BonFIRE spectra of other dyes. (a) ω_{IR}-dependence in 2D-BonFIRE for Cy5.5, ATTO665, ATTO680 and ATTO725. (b-e) ω_{probe}-dependence in 2D-BonFIRE for (b) Cy5.5 (structure inset), (c) ATTO665 (structure not publicly known), (d) ATTO680 (structure inset), and (e) ATTO725 (structure not publicly known; ω_{IR} = 1590 cm^{-1} and 2230 cm^{-1}). (f) ω_{IR}-dependence in 2D-BonFIRE for MgPc and ICG-d_7 (structures inset).

(g-h) ω_{probe}-dependence in 2D-BonFIRE for (g) MgPc and (h) ICG-d_7. Vertical dashed lines mark the specified energy gap from the UV-vis absorption maximum. The UV-vis spectrum of MgPc was obtained from the literature.[11]

One possible explanation for these shifts is that the f_{FC} (i.e., symmetry) of the IR-excited mode selects for different states in the S_1 manifold than the ground vibrational state. In this case, the different favored transitions could vary slightly between the nitrile and ring-breathing modes, leading to different but small blueshifts. Another possible explanation is thermochromism (heat-induced shifts in λ_{max}). Because DMSO is highly transparent at ω_{IR} = 1590 cm^{-1} and 2230 cm^{-1} and the dyes are fairly dilute, we estimate that the dominant source of heating in solution is water (hygroscopically absorbed from the atmosphere into the DMSO stock), where there is appreciable absorption from the OH-bend at 1635 cm^{-1} (leading to a larger shift) and a weaker absorption from the bend-libration combination mode of water near ~2100 cm^{-1} (leading to a smaller shift). Indeed, shifts of this magnitude (~150 cm^{-1}) have been previously observed in the context of elevated vibrational temperatures by Kaiser and co-workers.[8, 12]

Three strong bands were observed in the IR for MgPc (**Fig. S3f**), but ω_{probe} spectra could only be measured in the low-frequency tail due to the cutoff of the bandpass filter and dichroic mirror that were used (750 nm; **Fig. S3g**). For ICG-d_7, the strongest peak (ω_{IR} = 1470 cm^{-1}) obeys the resonance condition well (**Fig. S3h**). As with ATTO725, we observe slight blueshifts in the resonance condition for the 1390 and 1350 cm^{-1} modes of ICG-d_7, which may again be the result of elevated temperature (potentially from the CH-bending bands of DMSO), but it should be noted that ICG-d_7 exhibited generally worse SNR, despite being measured at 1 mM. The absence of a clear peak at ω_{IR} = 1560 cm^{-1} is thus attributed to lower SNR.

S3. Non-degenerate resonance-enhanced two-photon absorption.

There are three main sources of background in 2D-BonFIRE: anti-Stokes fluorescence, photothermal effects on fluorescence, and fluorescence following non-degenerate resonance-enhanced two-photon absorption (NDR-TPA; **Fig. S4**). We have described anti-Stokes fluorescence and photothermal effects in depth elsewhere, and their mechanisms are well-established.[1, 2] However, 2D-BonFIRE has provided new understanding of NDR-TPA, which we discuss here.

NDR-TPA is the virtual state-mediated alternative to BonFIRE (**Fig. S4a**), where the IR and probe photons are absorbed instantaneously for an electronic excitation ("non-degenerate" because $\omega_{IR} \neq \omega_{probe}$). NDR-TPA cross-sections are resonance-enhanced, leading to much stronger absorptions compared with traditional TPA.[13] In the fingerprint region, NDR-TPA is negligibly small compared to BonFIRE ($\sim 10^{-3}$). However, in the cell-silent and CH-stretching regions, BonFIRE is ~100-fold weaker, largely as a consequence of lower IR cross-sections, and thus NDR-TPA becomes more pronounced relative to BonFIRE. NDR-TPA is extremely important to consider because it sets the effective minimum BonFIRE cross-section for BonFIRE signal to still be measurable. For a mode with a short lifetime where BonFIRE is much weaker than NDR-TPA, BonFIRE signal may not be detectable with our current temporal resolution.

Kaiser and co-workers were the first to observe NDR-TPA, but they had initially attributed it to a continuum of combination bands and overtones with immeasurably short lifetimes.[14] With picosecond pulses, exceedingly short vibrational lifetimes are indistinguishable from NDR-TPA (though by the same logic, a resolvable vibrational lifetime with picosecond pulses is an unimpeachable indicator of a real-state intermediate).[15] It was not until Tokmakoff and co-

workers' studies with femtosecond pulses and improved temporal resolution that these signals could be confidently identified as NDR-TPA.[16]

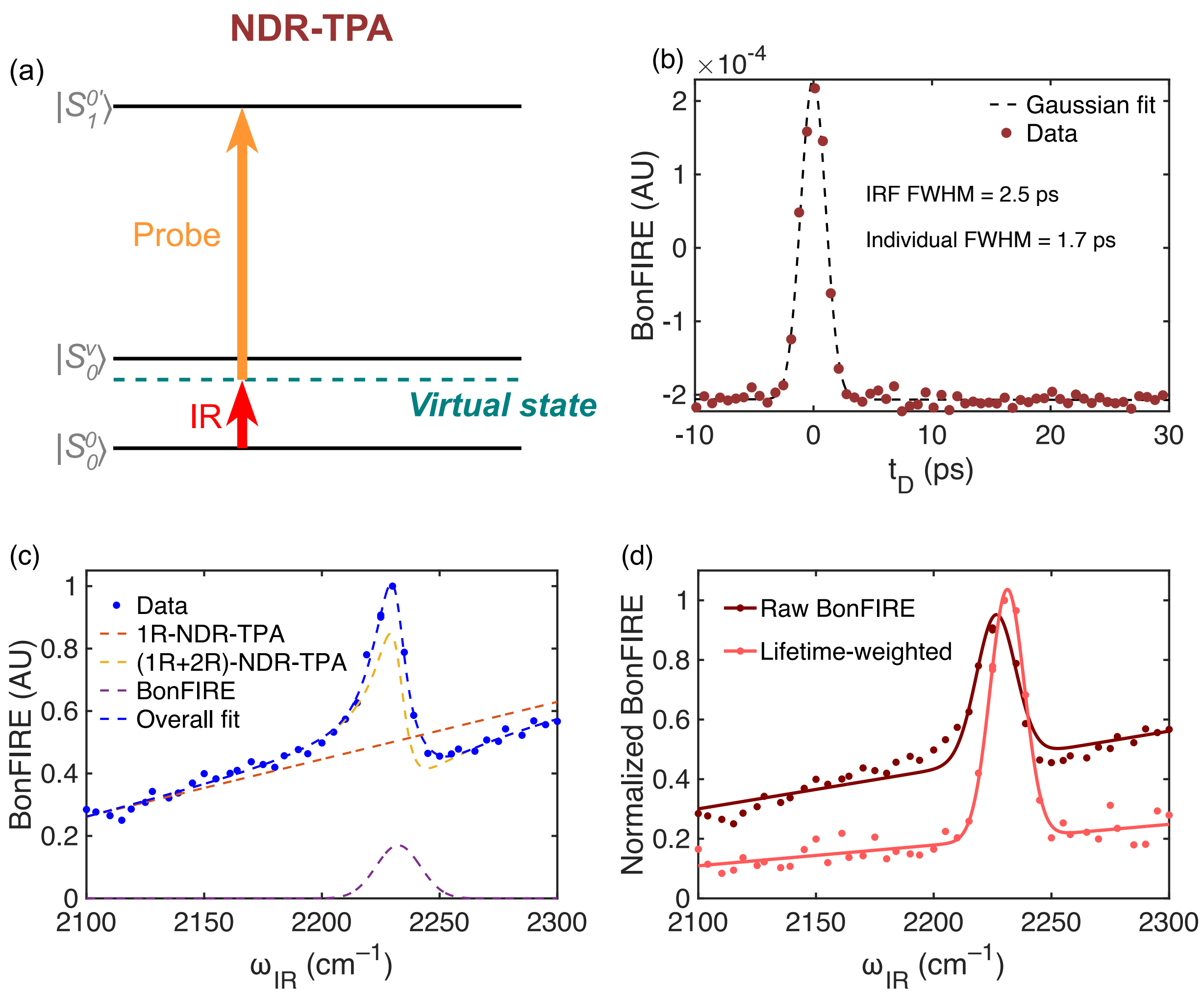

Figure S4. Non-degenerate resonance-enhanced two-photon absorption. (a) Energy-level visualization of NDR-TPA. (b) Direct characterization of the IRF by NDR-TPA at ω_{IR} = 3140 cm^{-1} and ω_{probe} = 11,550 cm^{-1}. The FWHM of the Gaussian fit is 2.5 ps, meaning the average pulse width of the IR and probe pulses is 1.7 ps. (c) ω_{IR}-dependence of NDR-TPA. Both singly resonant (1R)- and doubly resonant (2R)-NDR-TPA appear to be present, where 2R-NDR-TPA is modeled as a Fano resonance. (d) Background-free BonFIRE by vibrational lifetime-weighting. Given that NDR-TPA has no vibrational lifetime, weighting the BonFIRE spectrum by the best-fit vibrational lifetime at each frequency allows for effective removal of NDR-TPA, thereby narrowing and mitigating the distortion of the lineshape (visible by Gaussian fitting with a linear baseline).

As cleverly demonstrated by Tokmakoff and co-workers,[16] NDR-TPA can be used to directly characterize our IRF because the decay of the virtual state is a Dirac delta function ($IRF * \delta(t_D) = IRF$). As an example, we use ω_{IR} = 3140 cm^{-1} and ω_{probe} = 11,550 cm^{-1} to measure our IRF (**Fig. S4b**), obtaining an average pulse width of 1.7 ps for the IR and probe pulses. This result is in good agreement with our measured value of 1.6 ± 0.2 ps, which is the mean ± one standard deviation obtained from convolution fitting across the entire 2D-BonFIRE dataset.

Hales et al. experimentally and theoretically characterized NDR-TPA for electronic transitions in fluorene derivatives.[13] They show that the NDR-TPA cross-section (δ) is well-described as $\delta_T \approx \left\{\frac{3L_1^2L_2^2}{5n_1n_2c^2\varepsilon_0\hbar}\right\}\left[\frac{(\hbar\omega_1)^2\hbar\omega_2}{\hbar\omega_1+\hbar\omega_2}\right]\frac{\mu_{ge}^2\mu_{ee'}^2}{\Gamma_{ge}}\left(\frac{1}{E_{eg}-\hbar\omega_1}+\frac{1}{E_{eg}-\hbar\omega_2}\right)^2$ to include the effect of an intermediate state resonance-enhancement.[13] Crucially, the term in parentheses describes the frequency-dependence of the resonance enhancement, and the term in square brackets describes the *non-resonant* frequency-dependence of the NDR-TPA cross-section. In our case, $\omega_1 = \omega_{probe} \approx \frac{E_{eg}}{\hbar}$ (the near-resonant frequency) and $\omega_2 = \omega_{IR}$ (which in the baseline is far from resonance, compared to the linewidth of the transition). Given that $\omega_{probe} \gg \omega_{IR}$, the non-resonant frequency dependence $\left[\frac{(\hbar\omega_{probe})^2\hbar\omega_{IR}}{\hbar\omega_{probe}+\hbar\omega_{IR}}\right]$ simplifies to $[\hbar\omega_{probe}\hbar\omega_{IR}]$, showing that NDR-TPA is predicted to increase linearly with ω_{IR} and ω_{probe}. This linear dependence is exactly what we observe experimentally (linearly increasing with ω_{IR}; **Fig. 2d**, **Fig. S4c**), confirming the assignment of this linear background as singly resonant (1R-) NDR-TPA. This non-resonant frequency dependence also explains why bluer dyes exhibit larger NDR-TPA background, since they are necessarily measured with larger values of ω_{probe}.

We additionally wondered about the potential for doubly resonant (2R-) NDR-TPA, where ω_{IR} is near-resonant for the nitrile transition (in addition to ω_{probe} being near-resonant for the

electronic transition). Upon close examination, we observe that the lineshapes are visibly asymmetric (**Fig. S4c**), exhibiting a dispersive lineshape that is reminiscent of a Fano resonance (similarly observed by Tokmakoff and co-workers).[16] This dispersive lineshape could plausibly be due to 2R-NDR-TPA, although it is not currently clear why the lineshape is dispersive rather than absorptive (the vibrational resonance may be coupled to the imaginary part of $\chi^{(3)}$; similarly postulated by Whaley-Mayda). Regardless, the dispersive lineshape results in a slight redshift of the frequency where maximal intensity is observed, suggesting that the raw BonFIRE spectrum is distorted by NDR-TPA.

Given that NDR-TPA exhibits no vibrational lifetime (**Fig. S4b**), we reasoned that weighting the spectrum by the vibrational lifetime measured at each frequency could allow for effective removal of both 1R- and 2R-NDR-TPA. As shown in **Fig. S4d**, lifetime-weighted BonFIRE restores both the peak position and lineshape, due to removing the dispersive distortion. However, it still results in a nonzero baseline offset because fitting of NDR-TPA with a floated lifetime generally yields a short but nonzero best-fit lifetime (e.g., 0.3 ps). We note that lifetime-weighting works better with longer lifetimes and is particularly effective for nitriles (monoexponential decays) due to their less strict SNR requirements for accurate fitting.[2]

S4. Additional evidence of combination modes in 2D-BonFIRE.

Sakai and co-workers have shown in jet-cooled supersonic expansions that the excitation of a CH- or NH-stretching mode (low f_{FC}, high σ_{IR}) and subsequent IVR into high-f_{FC} modes is possible.[17] In such a case, double-resonance signal rises at a later time, following vibrational relaxation by IVR. Conversely, the rise-time should remain unchanged for a combination mode, where the high-f_{FC} mode is excited immediately.[17]

In our experiments, the rise-time of BonFIRE (**Fig. S5a**; black curve) is not delayed relative to NDR-TPA (**Fig. S5a**; red and blue curves, which show no vibrational lifetime). As further validation, anharmonic DFT calculations[2] predict many (surprisingly strong) IR-active combination modes in the CH-stretching region (**Fig. S5b**) that are absent in harmonic DFT. We emphasize that the plotted data are *not the total* anharmonic density of states, but the predicted IR absorption spectrum (i.e., *only the IR-active* combination modes) at the lowest anharmonic order.

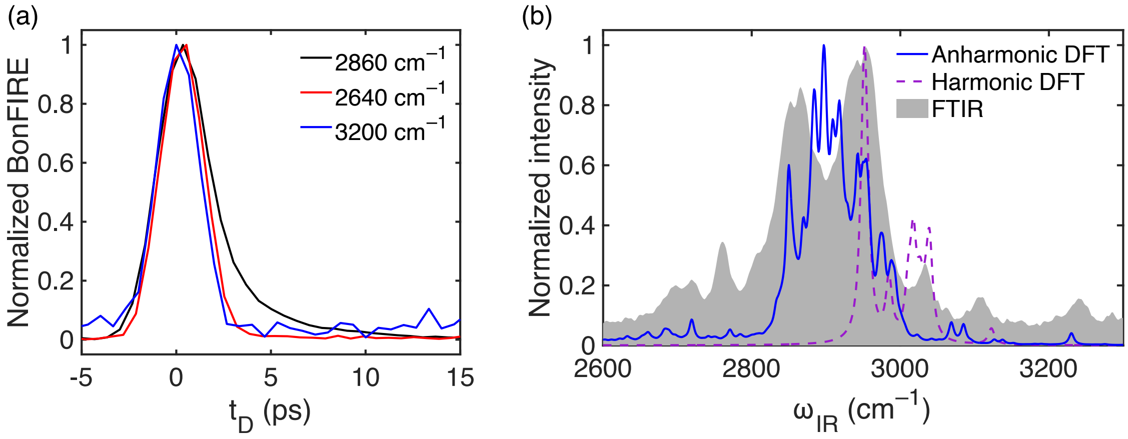

Figure S5. BonFIRE rise-time in combination modes and anharmonic DFT. (a) BonFIRE rise-time. BonFIRE (black) is not visibly delayed relative to NDR-TPA (red and blue) at ω_{probe} = 12,120 cm^{-1}. Time delays are corrected for laser tuning (see **Section S1**). (b) Predicted IR absorption by anharmonic DFT for Rh800 in the CH-stretching region. Many combination modes are predicted to be strongly IR-active.

From the above data and rationale, we believe it is more reasonable that the observed signals in the CH-stretching window (2600-3200 cm^{-1}) result from IR-excited combination modes rather than CH-stretches. We further argue this case here with additional supporting experiments. First, we consider the case of a deuterated dye. If the observed signals in the 2600-3200 cm^{-1} region originated from CH-stretches, then we would expect BonFIRE from CD-stretches in a deuterated dye. Instead, if the observed signals originated from combination modes, then we would expect no BonFIRE signal in the CD-stretching window. The fully deuterated analogue of Rh800 is not commercially available, nor is it trivial to synthesize. Instead, we used ICG-d$_7$ as a control and attempted BonFIRE in the CD-stretching window (2100-2600 cm^{-1}; **Fig. S6**).

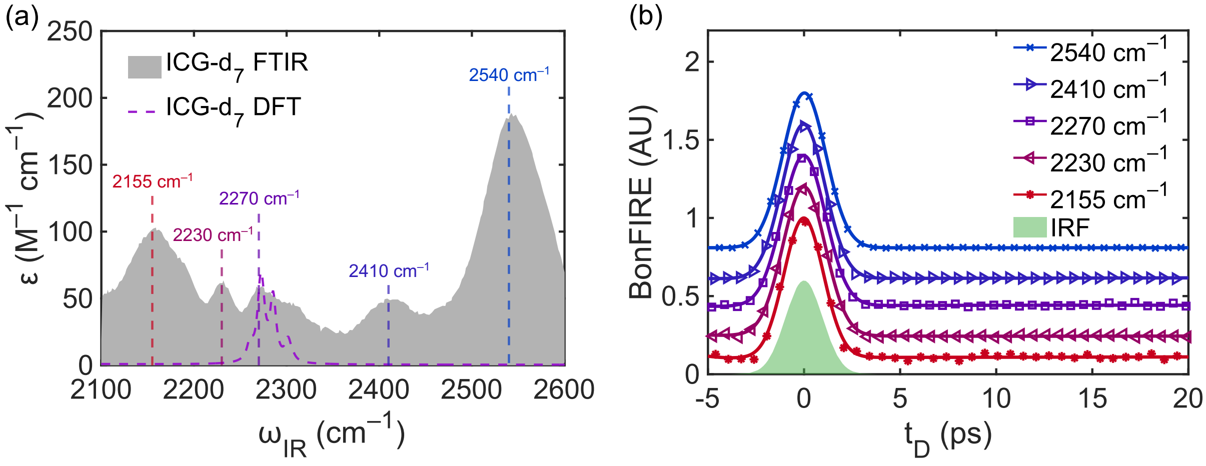

Figure S6. Absence of CD-stretching BonFIRE in ICG-d$_7$. (a) FTIR spectrum of ICG-d$_7$ and harmonic DFT (scaled by 0.975). A few weak peaks are observed, including an apparent CD-stretching band at 2270 cm^{-1}. (b) BonFIRE attempts at several values of ω_{IR} in the CD-stretching window (vertically displaced for clarity). NDR-TPA is consistently detected (signal identical to the Gaussian IRF, plotted in green), but no signal with a discernible vibrational lifetime is observed.

While we observe multiple peaks in FTIR (most notably, the 2270 cm^{-1} peak, which appears to correspond to a CD-stretch by comparison with DFT; **Fig. S6a**), only NDR-TPA is

observed in the time domain (reproducing the IRF; **Fig. S6b**), with no BonFIRE present (i.e., no discernible vibrational lifetime). Thus, pumping a CD-stretch in a deuterated dye does not result in BonFIRE, further supporting that the observed signals in the CH-stretching window originate from combination modes.

We further note that we were indeed able to observe a delayed rise-time in the low-frequency fingerprint region (**Fig. S7**; 1100 cm^{-1} mode, temporally referenced to the 1200 cm^{-1} excitation). The 1100 cm^{-1} mode of Rh800 is not part of our current survey, but it does provide direct experimental confirmation that we can observe a delayed rise-time, even with 1.6-ps pulses. Ultimately, resolving such a delay is a function of SNR. In fact, we have shown previously that our system could resolve vibrational lifetimes as short as 0.2 ps with 99% accuracy, owing to the high SNR of our data[2] (our measurements in the CH-stretching window feature SNR typically >100, as shown in **Fig. S5**).

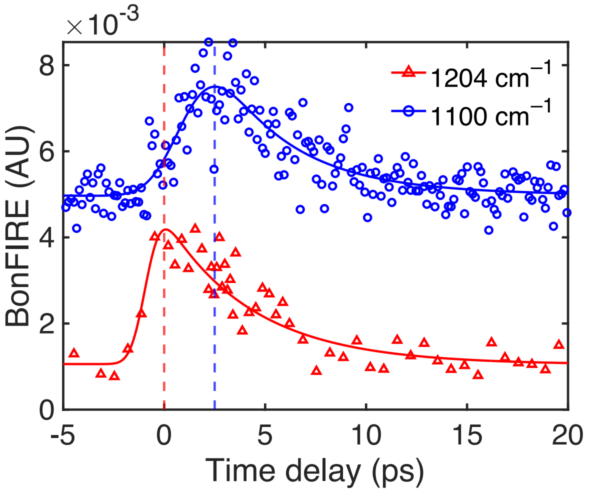

Figure S7. Delayed rise-time at 1100 cm^{-1}. The 1100 cm^{-1} mode of Rh800 exhibits a delayed rise-time, indicating that the IR-excited state is not FC-active, but it decays into an FC-active state (data are vertically offset for clarity).

One may still argue that the 1100 cm^{-1} mode may decay *slowly* into its BonFIRE-active state (and thus we can resolve it), but the CH-stretches may decay almost instantaneously (and thus we would be unable to resolve a delayed rise-time). However, this argument is inconsistent with both Sakai and co-workers' data (where the rise-time was delayed by ~1 ps for a CH-stretch)[17] and the observed ω_{probe}-dependence in 2D-BonFIRE. As we describe further in **Section S5**, IVR in large molecules can be conceptually described as a randomization of the excited vibrational quanta towards a Boltzmann distribution. Because the vibrational energy stays in the molecule, the Boltzmann distribution is of an elevated, 'hot' vibrational temperature (T^*).[15] As such, the ω_{probe}-dependence after IVR is well-modeled as an exponential ($\sigma_{probe} = \sigma_{00} \cdot \exp\left(\frac{100hc(\omega_{probe}-\omega_{00})}{k_B T^*}\right)$), effectively describing UV-vis absorption at frequencies below the 0-0 transition.[15]

We next experimentally confirm this interpretation by visualizing our ω_{probe}-dependence as a function of t_D, as plotted in **Fig. S8** (all traces are normalized). We first examine the ω_{IR} = 1440 cm^{-1} data (**Fig. S8a**). The peak at ω_{probe} ~ 12,800 cm^{-1} (in the t_D = 0 ps red curve) decays first, reaching a roughly exponential curve at t_D = 6 ps (gray curve) that is still elevated above the baseline. The presence of a peak indicates that a specific mode is preferentially populated above its thermal baseline (i.e., a specific mode was excited by the IR pulse). Correspondingly, the disappearance of the peak is due to IVR, indicating that the specific mode populated by the IR excitation has been depleted. The second, slower decay is VC, where the hot Boltzmann distribution transitions to a distribution of lower T^* by dissipating energy to the solvent bath.

Convincingly, an analogous trend is observed in the 2860 cm^{-1} data (**Fig. S8b**). If the BonFIRE signal at 2860 cm^{-1} resulted from a hot Boltzmann distribution after rapid IVR (faster than for what we can observe a delayed rise-time), then there should be no peak in **Fig. S8b** at t_D = 0 ps. Instead, the presence of a peak confirms that a specific vibrational population exists.

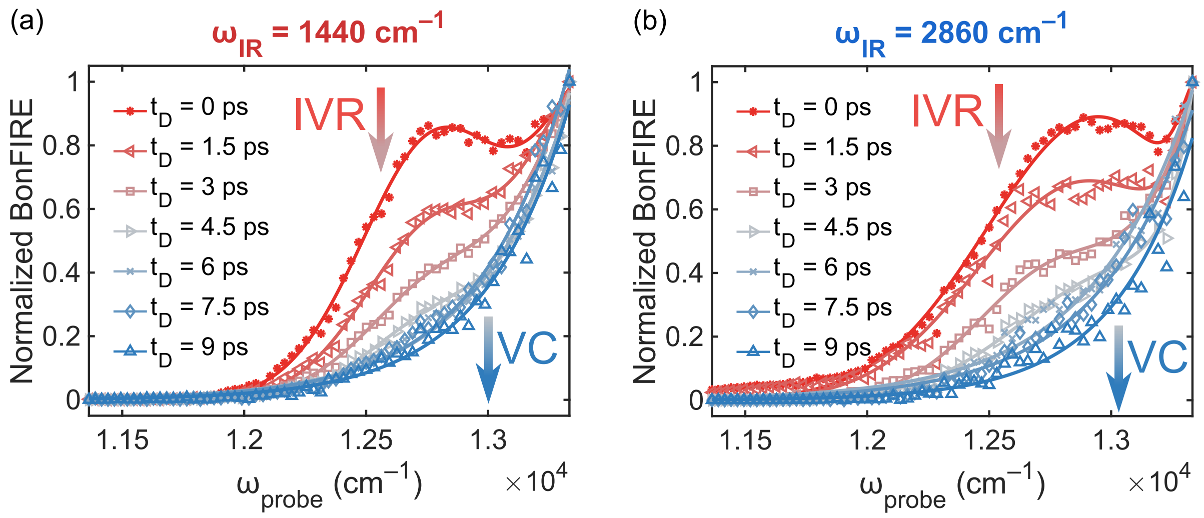

Figure S8. Time-evolution of probe-dependence in 2D-BonFIRE. (a-b) ω_{probe} traces at varying t_D for (a) 1440 cm^{-1} and (b) 2860 cm^{-1} (normalized to the point of maximum intensity). The visible presence of a peak is confirmation that a specific mode is populated. The decay of the peak towards a raised exponential curve is due to IVR. The transition of the raised exponential toward a lower one is due to VC.

To summarize, we provide a visual comparison of the five major possible cases of BonFIRE in the 2600-3200 cm^{-1} region in **Fig. S9**. We note that Case I was reported by Kaiser and co-workers for the NH-stretch of Coumarin 7,[15] and either Case III or IV describes Sakai and co-workers' experiments.[17] Considering our 2D-BonFIRE dataset as a whole, it is most reasonable that the observed violations of the resonance condition are due to the direct excitation of combination modes (Case II). Mechanistically, we expect that this resonance-breaking results from mechanical anharmonicity rather than electrical anharmonicity, which should be the dominant source of anharmonicity in polyatomic molecules.[18]

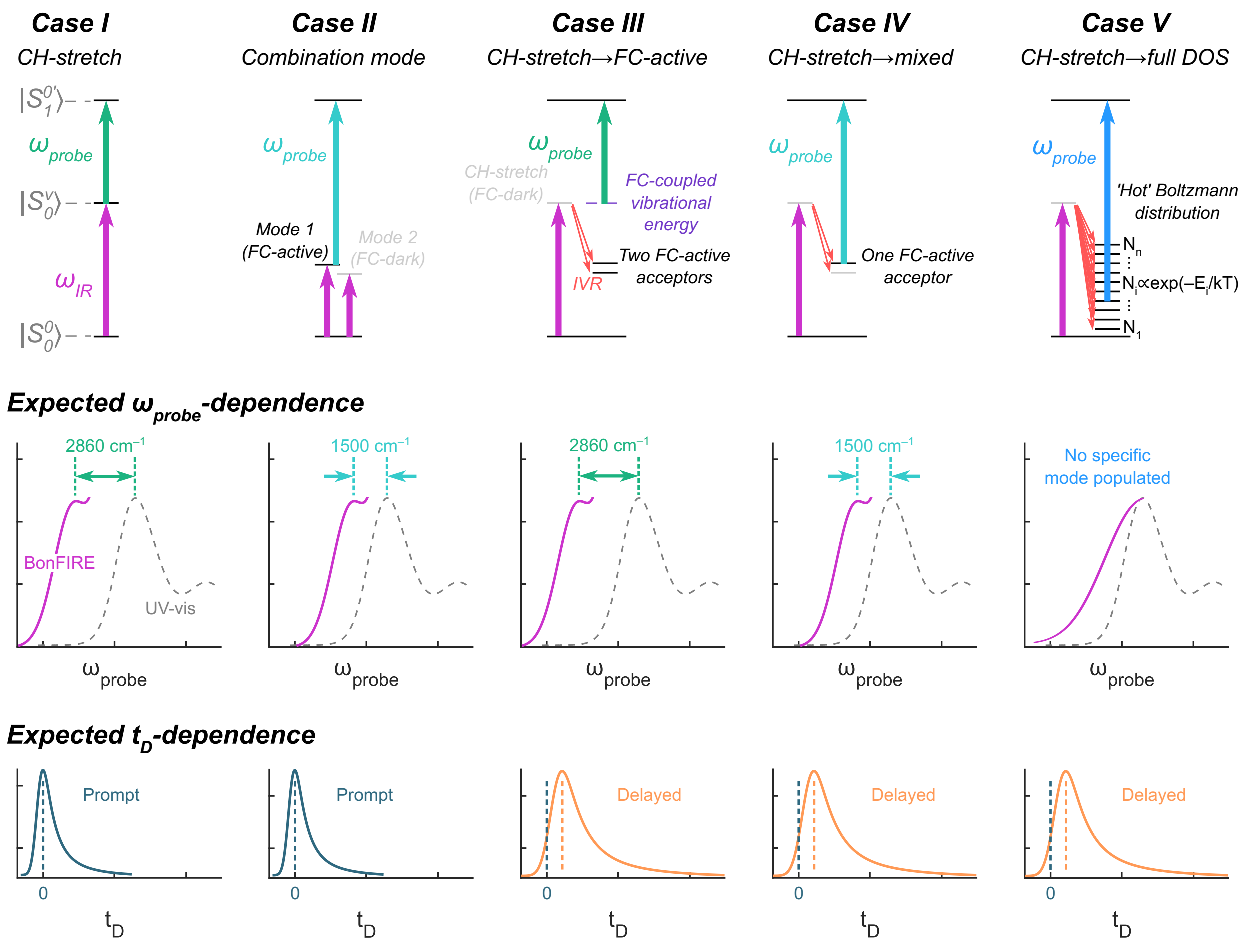

Figure S9. Comparison of BonFIRE signal mechanisms. Five possible cases in the CH-stretching region are considered, which each exhibit characteristically different signals in the frequency and time domains. Of these five cases, only Case II (the combination mode) accurately describes what is observed in 2D-BonFIRE in the 2600-3200 cm^{-1} region with Rhodamine dyes.

Finally, to validate the generality of our findings, we measured spectra of ATTO665, ATTO680, ATTO725, Cy5.5, ICG, and ICG-d_7 in the CH-stretching region (**Fig. S10**). Interestingly, we observe that the xanthene/rhodamine-based dyes all exhibit peaks that appear to be combination modes (**Fig. S10a-d**), but the cyanine-based dyes exhibit only a broad background from NDR-TPA (**Fig. S10a**). This clear trend suggests a fundamental difference in the vibrational

structures of these dyes. We note that strong combination bands and overtones are common in benzene (i.e., the famous "benzene fingers"), xanthene,[19] and other aromatic molecules.[20, 21]

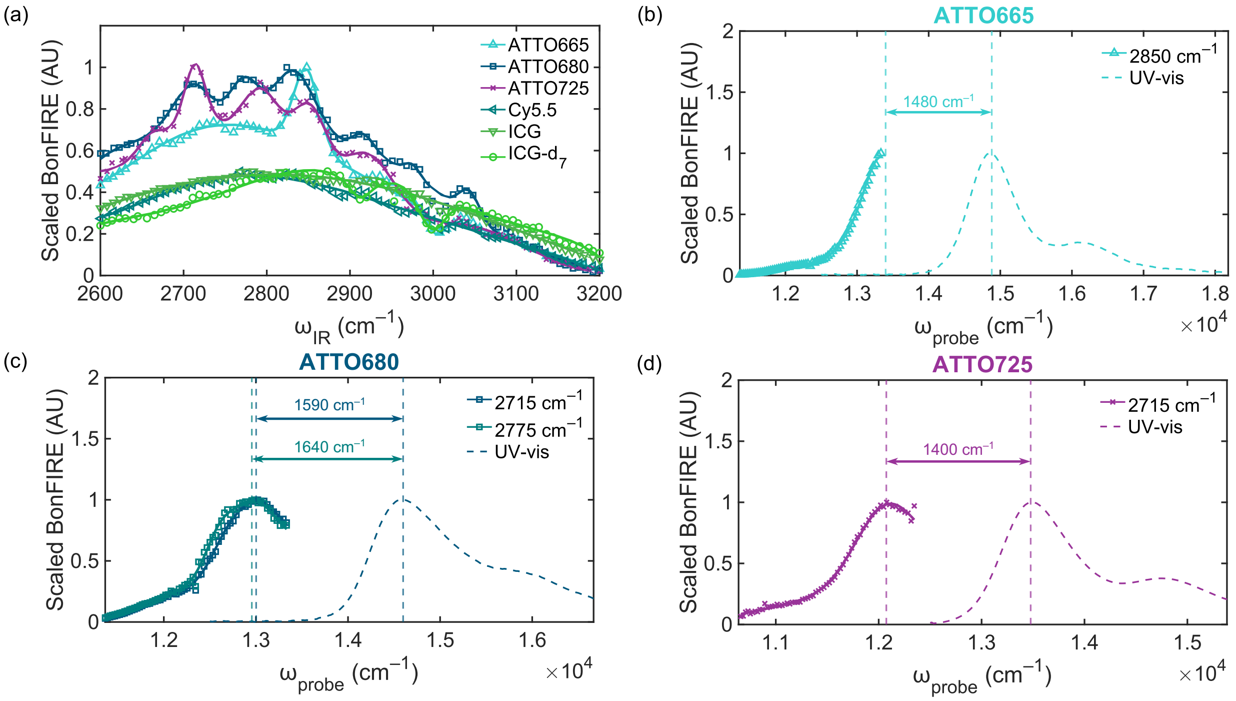

Figure S10. Combination modes in 2D-BonFIRE with other dyes. (a) BonFIRE ω_{IR}-dependence in the CH-stretching region for ATTO665, ATTO680, ATTO725, ICG, ICG-d$_7$, and Cy5.5. The dips at ~2912 cm^{-1} and ~2996 cm^{-1} are due to DMSO absorption (samples were diluted into DMSO-d$_6$ for measurement, but the stocks were dissolved in DMSO). (b-d) BonFIRE ω_{probe}-dependences in the CH-stretching region for (b) ATTO665, (c) ATTO680, and (d) ATTO725. As with Rh800, each of these modes shows violation of the resonance condition.

S5. Vibrational relaxation in 2D-BonFIRE.

The elongated fingerprint decays observed at larger ω_{probe} can be qualitatively rationalized by **Fig. S2**, using the simple picture that up-conversion becomes possible from decayed states. However, this picture must be elaborated to account for the sigmoidal trend in A_1/A_2 in the ATTO665/680/725 dye series. To explain, we picture an ensemble of molecules occupying a continuum DOS (**Fig. S11**).[22] The IR-excited mode in the molecule decays rapidly through IVR (~1 ps; **Fig. S11a**).[2] Across the ensemble of molecules, the energy is statistically redistributed across the many near-isoenergetic modes, facilitated as third-order decays mediated by low-frequency (<200 cm^{-1}) “phonon” modes (i.e., anharmonic mixing with thermally populated modes).[2] Following IVR (e.g., at t_D = 6 ps),[15] the IR-excited state is effectively depleted, and the distribution of vibrational energy is described as a Boltzmann distribution of an elevated vibrational temperature (**Fig. S11b**).[23] Crucially, IVR does not reduce the total vibrational energy of the molecule; it only redistributes the energy into different (generally, lower-energy) vibrational modes.

Subsequently, this ‘hot’ Boltzmann distribution cools towards a room-temperature Boltzmann distribution (hence the name “vibrational cooling”), where energy leaves the molecule and is dissipated into its surroundings (**Fig. S11a**, **b**). The precise mechanism of VC initially proved elusive, with early theories including through-bond IVR[14] and collision-induced energy transfer to the solvent.[15, 24] Ultimately, Dlott and co-workers provided convincing evidence that phonon-mediated intermolecular vibrational energy transfer to the solvent is the mechanism of vibrational cooling of the IR-excited dye (and thereby, vibrational heating of the solvent bath, through the mechanism they termed “multiphonon up-pumping”).[25, 26]

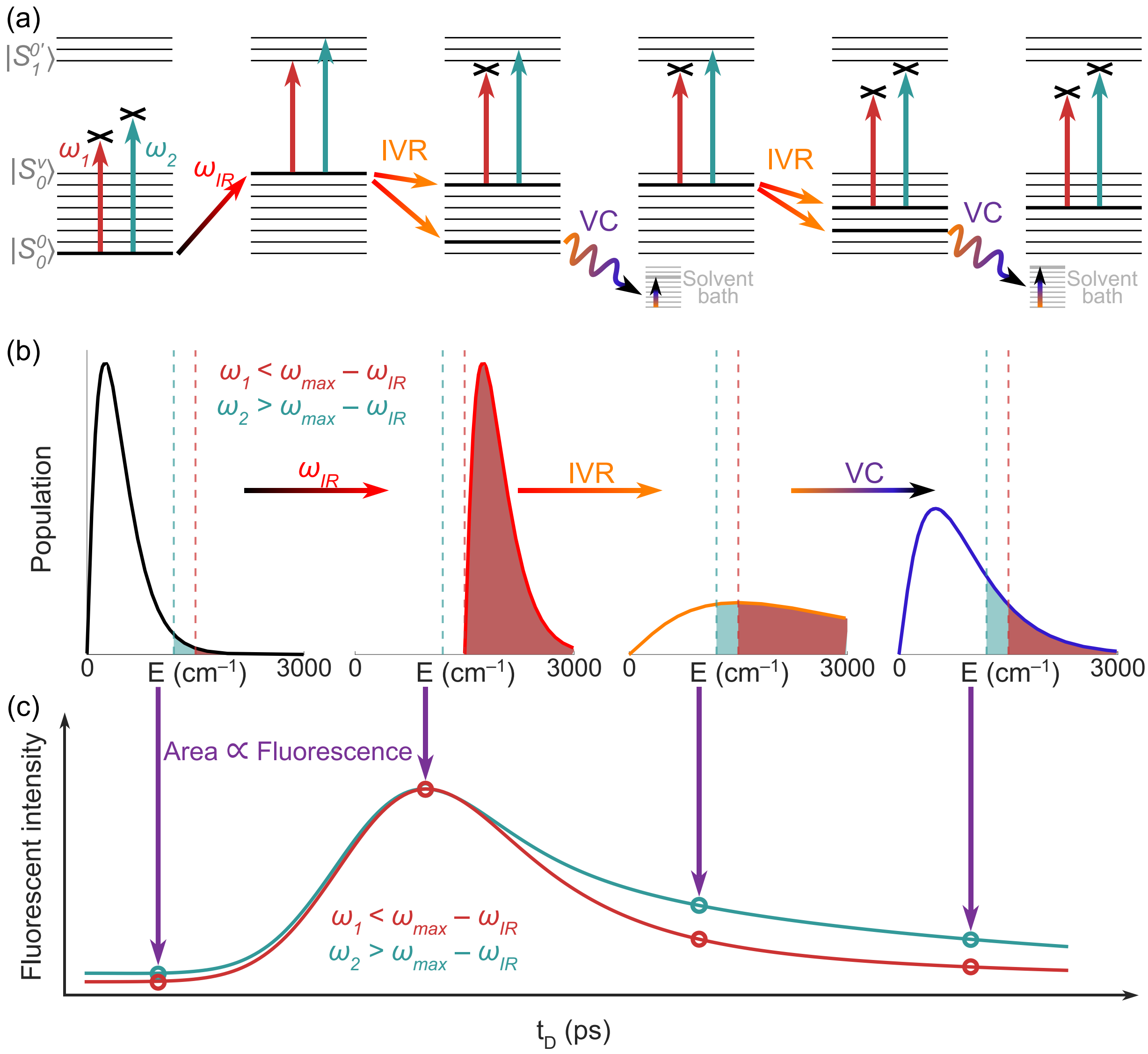

Figure S11. Mechanistic picture of vibrational relaxation. (a) Visualization of cascaded vibrational relaxation within a single molecule at two different ω_{probe} (ω_1 and ω_2). Occupied vibrational modes are bolded. "X" marks placed above arrows indicate insufficient energy for up-conversion. In this visualization, the low-frequency modes are assumed to not contribute to double-resonance. (b) Boltzmann population curves through excitation, IVR, and VC.[8] (c) Fluorescent intensity in BonFIRE as a function of time delay. Fluorescence is proportional to the area under the Boltzmann population curve from $\omega_{00} - \omega_{probe}$ to infinity (shaded blue or red correspondingly by ω_{probe}). Because a larger fraction of the population is probed as ω_{probe} increases, the fluorescent decay slows, even though the underlying vibrational dynamics are unchanged.

Finally, we must consider that signal in BonFIRE is integrated fluorescent intensity (**Fig. S11c**), which is proportional to the population of molecules with sufficient energy to be up-converted to S_1 (shaded areas under the Boltzmann curves in **Fig. S11b**). Visibly, it is clear that a larger fraction of the population is excited for larger ω_{probe} after IVR and VC (i.e., the area right of the blue dashed line is larger than the area right of the red dashed line). Though it is less obvious visually, it can also be seen that for larger ω_{probe}, the area under the curve *changes less* from IVR. Physically, this difference reflects that A_1/A_2 decreases as ω_{probe} increases. Of course, to provide a quantitative model for A_1/A_2 would require more detail (e.g., the calculation of f_{FC} for each state, which are treated as equal in this Boltzmann picture). However, this illustration serves as a useful visualization of vibrational relaxation and how it is probed in 2D-BonFIRE as a function of ω_{probe}.

S6. Vibrational cooling rate dispersion in 2D-BonFIRE.

For the strongest modes of Rh800 (1300 cm^{-1}, 1500 cm^{-1}, and 1590 cm^{-1}), we can confidently observe deviations from pure biexponential character, evidenced by the oscillations in the residuals of the fit (**Fig. S12a-b**; **Fig. 3h**). These deviations are most obvious for the 1300 cm^{-1} mode, since it features the slowest overall decay (**Fig. S12a**). The oscillations in the biexponential residuals additionally develop on different timescales, indicating a molecular response rather than an instrumental artifact (where in contrast, the oscillations in the pulse-overlap region around t_D = 0 ps are more consistent and likely indicate deviation from ideal Gaussian pulse shapes). The presence of VC rate dispersion was confirmed to be independent of t_D step size, IR modulation scheme, optical power, and slight differences in ω_{IR} within a given vibrational band, and these trends were observed on several different days.

It is worth noting that the SNR requirements for accurate biexponential fitting with VC dispersion are quite stringent. For monoexponential decays (e.g., nitriles) on the order of 0.8-1.2 ps, we previously established that an SNR of ~50 was sufficient for accurate fitting.[2] Applying the same simulation methods to pure biexponential decays,[2] we find that the requisite SNR is ~500, meaning fitting a biexponential requires an order of magnitude better SNR than a monoexponential. As A_1/A_2 diverges from 1, the SNR requirements become stricter, since one component becomes weaker and thus harder to resolve. Inclusion of VC rate dispersion in the simulations yields even more challenging fitting, requiring SNRs > 1100 for > 98% accurate fitting across $\beta \in [0.5, 1.5]$. These fits were also much more sensitive to their initial conditions. Notably, smaller values of β impose slightly less strict SNR requirements, due to the overall decay being 'stretched' longer for $\beta < 1$.

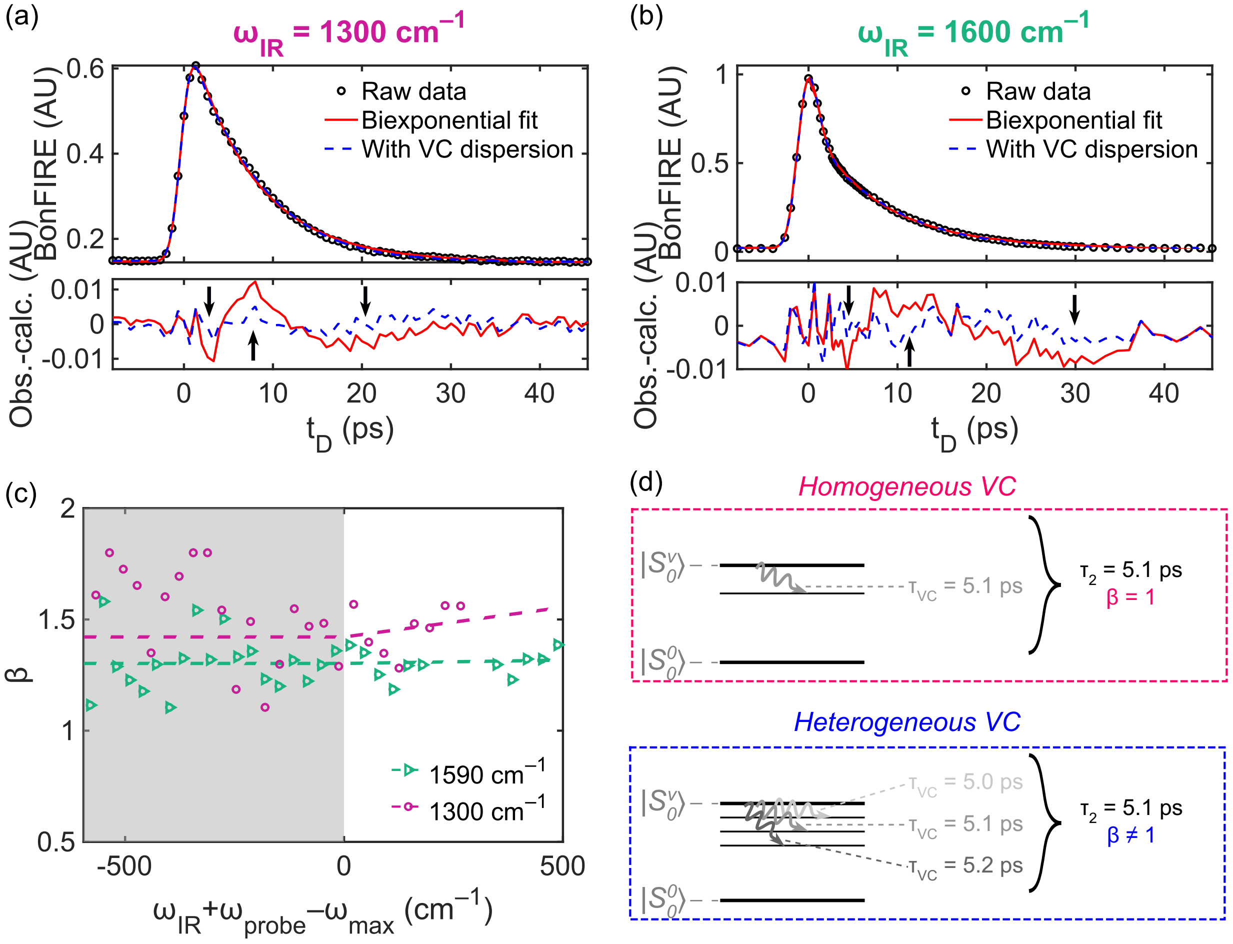

Figure S12. Vibrational cooling rate dispersion in other vibrational modes. (a-b) VC rate dispersion in the (a) 1300 cm^{-1} and (b) 1590 cm^{-1} modes of Rh800 at ω_{probe} = 13,330 cm^{-1}. (c) β as a function of excitation energy for 1590 cm^{-1} (green) and 1300 cm^{-1} (pink). (d) Conceptual illustration of homogeneous and heterogeneous VC. In the drawn example, the heterogeneity results from the presence of multiple possible VC pathways. As a reminder, VC involves the transfer of a small amount of energy (e.g., ~100 cm^{-1}) to the solvent bath (**Fig. S11**).

For the 1590 cm^{-1} and 1300 cm^{-1} data, the SNRs are ~300, meaning the obtained fits carry an inherent error of at least ± 10%, and no clear trend is visible (**Fig. S12c**). Furthermore, as ω_{probe} decreases, VC dispersion eventually disappears below the noise floor (**Fig. S12c**). These demanding SNR requirements also justify why such features have not been previously reported (to

our knowledge) in 1DVF literature. BonFIRE, with picosecond pulses at 80 MHz, appears uniquely suited to the study of individual vibrational mode dynamics with rapid averaging.

At 1500 cm^{-1}, our peak SNR is ~1200, meaning that an accurate trend can be obtained. For this mode of Rh800, we observe that VC is more heterogeneous at low ω_{probe} and becomes more homogeneous as ω_{probe} increases (**Fig. 3i**), appearing to approach 1 (within experimental error) at large ω_{probe}. To help explain the mechanism behind this homogenization, we first consider a UV-vis absorption experiment measuring the conversion of two chemical species, A and B ($A \rightleftharpoons B$). If A and B have the same absorption spectrum and the same cross-section, then the absorption spectrum would show no change, regardless of the progress of the reaction. The key idea here is that a spectroscopic technique is only sensitive to processes that change the spectroscopic observable.

The same logic applies to the sensitivity of BonFIRE to VC (**Fig. S13**). For simplicity, we consider a single vibrational state with three possible VC pathways (**Fig. S13a**). At low ω_{probe}, all of these possible pathways lead to loss of BonFIRE signal (**Fig. S13b**). However, at larger ω_{probe}, some of the decayed states can be up-converted to S_1 (this is analogous to A and B having the same UV-vis absorption; the initial and decayed states are both BonFIRE-active). In this case, BonFIRE loses sensitivity to these pathways (the two lighter gray pathways; **Fig. S13c**), meaning that the observed decay becomes more homogeneous (i.e., BonFIRE is only sensitive to the dark gray pathway).

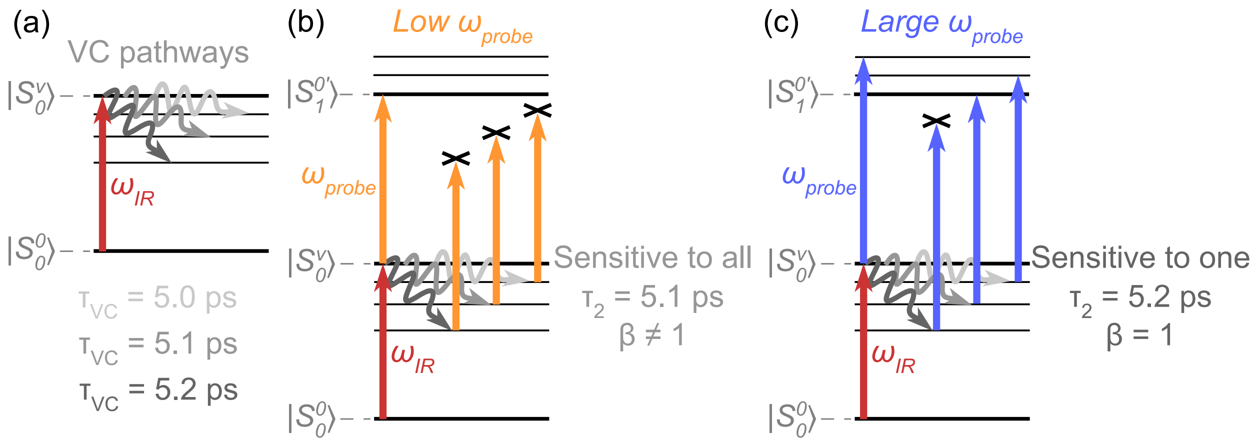

Figure S13. Conceptual illustration of VC rate dispersion in the 1500 cm^{-1} mode of Rh800. (a) Prototypical vibrational state with multiple possible VC pathways (occurring after IVR from the 1500 cm^{-1} mode). The different VC pathways result in losses of different amounts of energy to the solvent. (b) 2D-BonFIRE at low ω_{probe}. All three VC pathways lead to loss of signal, so BonFIRE is sensitive to all three pathways, leading to an overall heterogeneous decay. (c) 2D-BonFIRE at large ω_{probe}. Only the dark gray pathway leads to loss of signal, so BonFIRE is sensitive only to that pathway, leading to a homogeneous decay.

This model can rationalize our observed data at 1500 cm^{-1}, but it does not explain the observed VC dispersion at 1300 cm^{-1} and 1590 cm^{-1} in the high-ω_{probe} regime. In these cases, the observed heterogeneity may result from the probing of multiple vibrational energy levels (each with their own unique VC rate) rather than the heterogeneity of a single state with multiple pathways. This alternate mechanism would predict that the decay becomes more homogeneous as ω_{probe} decreases, but we currently do not have sufficient SNR to observe such a trend. However, it does seem reasonable that the observed heterogeneity at 1300 and 1590 cm^{-1} obeys a different mechanism than that observed at 1500 cm^{-1}, due to β being < 1 for 1500 cm^{-1}, but > 1 for 1300 and 1590 cm^{-1}. We suspect both mechanisms of VC dispersion are present simultaneously in all modes, with the dominant mechanism being dependent on the mode and its local DOS.

S7. Single-molecule sensitivity of 2D-BonFIRE.

It is worth noting that there is another possible two-pulse scheme for 2DVF (electronic pump, vibrational probe). However, this pulse scheme would likely be limited in sensitivity, as the fluorescence quantum yield is unlikely to change from vibrational excitation in the S_1 manifold except in the case of significant heating (as from an optically thick sample).[2] Thus, our pulse sequence in 2D-BonFIRE (vibrational pump, electronic probe) is currently the most promising avenue for pursuing single-molecule 2D vibrational spectroscopy.

To demonstrate the sensitivity of 2D-BonFIRE, we measured Rh800 in highly dilute solutions in DMSO. Spectra of the 1500 cm^{-1} mode at 1 nM (reproduced in two independent samples) and 100 nM are shown below in **Fig. S14**. In BonFIRE, the effective single-molecule concentration is 5 nM,[1] implying that 1 nM should be well into the single-molecule regime. As expected, the SNR is lower for dilute samples (~5 at 1 nM), but BonFIRE is still visibly apparent above the baseline noise in each scan.

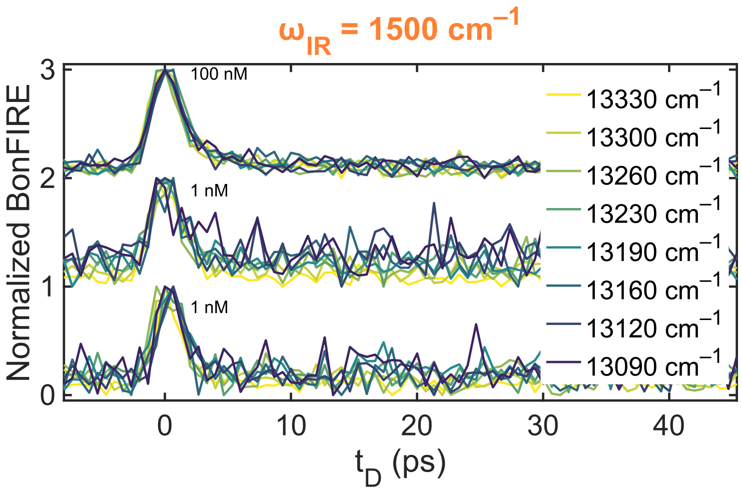

Figure S14. 2D-BonFIRE in highly dilute solutions of Rh800. Clear BonFIRE signal is visible at 1 nM (two independent samples) and 100 nM at varying ω_{probe}, demonstrating the sensitivity to see signals in solutions diluted to the level of single molecules.

Compared to our previous work, our PMT detection sensitivity has been improved by roughly one order of magnitude.[1] This improved sensitivity is attributed to several factors, including improvements to our confocal detection and optimization of the power outputs and pulse widths of our OPOs (now τ_p = 1.6 ± 0.2 ps instead of our previous 1.84 ps).[2] Additionally, we can now use a chopper to modulate our DFG pulse train while maintaining high powers, allowing us to observe saturation behavior for strong mid-IR transitions (**Fig. S15**). It is worth noting that the saturation that we observe here results from a balancing of the IR absorption rate and the spontaneous vibrational decay rate due to IVR (typically ~1 ps^{-1}).[2]

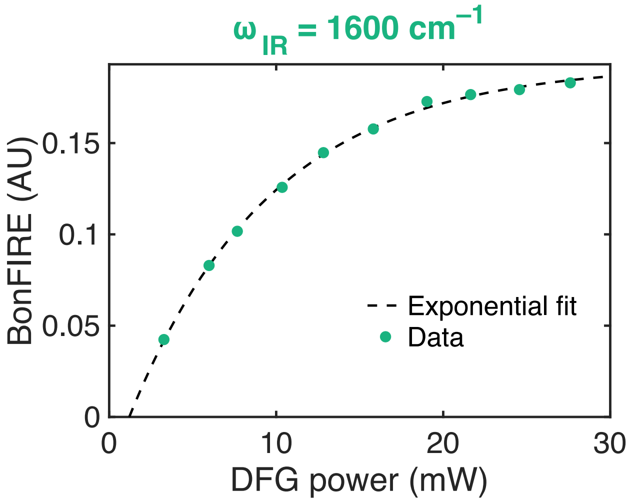

Figure S15. Saturated mid-IR absorption in 2D-BonFIRE. The DFG power-dependence of BonFIRE intensity fits well to an exponential with a vertical offset, indicative of saturated mid-IR absorption. Note that the x-axis denotes estimated power on-sample after the transmission losses of the ZnSe lens and the power loss due to chopping. These measurements were taken on 100 μM Rh800 in DMSO-d_6 with ω_{IR} = 1600 cm^{-1}, ω_{probe} = 12,820 cm^{-1} (780 nm), and 10 mW of probe power (similar behavior was observed at 1 mW, 0.1 mW, and 0.01 mW of probe power).

S8. Unmixing of hyperspectral 2D-BonFIRE images by CL+LASSO.

As mentioned in the main text, we imaged each polymer film at each combination of ω_{IR} = 2229, 2201, 2176, and 2148 cm^{-1} and ω_{probe} = 12,350, 11,930, 11,490, and 11,110 cm^{-1} (λ_{probe} = 810, 838, 870, and 900 nm). These IR frequencies were chosen as the means of the vibrational frequencies of the isotopologues, and these probe frequencies were chosen as points where the relative intensities of the different electronic scaffolds were characteristic. Specifically, BF2224 exhibits nearly the same BonFIRE intensity at 11,490 and 11,110 cm^{-1}, but BF2227's BonFIRE intensity decreases by roughly two-fold. A similar relationship applies for 11,930 and 11,490 cm^{-1} and distinguishing between BF2227 and BF2231. For BF2231 and BF2234, we could not measure the analogous points due to the cut-off wavelengths of our dichroic mirror and bandpass filter. However, between 12,350 and 11,930 cm^{-1}, BF2231's intensity decreases two-fold, but BF2234's intensity decreases four-fold. Thus, we reasoned that this set of frequencies was the minimum set necessary to distinguish between all 16 nitrile dyes.

To ensure that we captured the t_D-dependence of BonFIRE, we acquired images at t_D = –8.7, 0, 2.7, and 26.7 ps for each value of ω_{IR} and ω_{probe}. The –8.7-ps and 26.7-ps points allow us to assess baseline noise and background, the 0-ps point informs maximal BonFIRE intensity, and the point at 2.7 ps crucially allows us to differentiate between NDR-TPA (see **Section S3**) and BonFIRE. Therefore, our BonFIRE hyperstack comprises a set of 64 images, spanning each combination of 4 values of t_D, ω_{IR}, and ω_{probe}.

The unfolded hyperspectra (averaged across each individual film) are shown as one-dimensional data in **Fig. S16**, along with the remaining reference spectra not shown in the main text (**Fig. 4b-c**). Each group of 4 points corresponds to t_D = –8.7, 0, 2.7, and 26.7 ps. The first 4 points correspond to ω_{IR} = 2229 cm^{-1} and ω_{probe} = 12,350 cm^{-1}. Points 5-8 correspond to ω_{IR} =

2201 cm^{-1} and ω_{probe} = 12,350 cm^{-1}, points 9-12 correspond to ω_{IR} = 2176 cm^{-1} and ω_{probe} = 12,350 cm^{-1}, and points 13-16 correspond to ω_{IR} = 2148 cm^{-1} and ω_{probe} = 12,350 cm^{-1}. The same ordering of t_D and ω_{IR} holds for the next three groups of 16, but points 17-32 have ω_{probe} = 11,930 cm^{-1}, points 33-48 have ω_{probe} = 11,490 cm^{-1}, and points 49-64 have ω_{probe} = 11,110 cm^{-1}. Encouragingly, the differences between the hyperspectra are visible by eye (e.g., only the dyes with the reddest scaffold, BF2224/2196/2171/2143 have appreciable BonFIRE at ω_{probe} = 11,110 cm^{-1}).

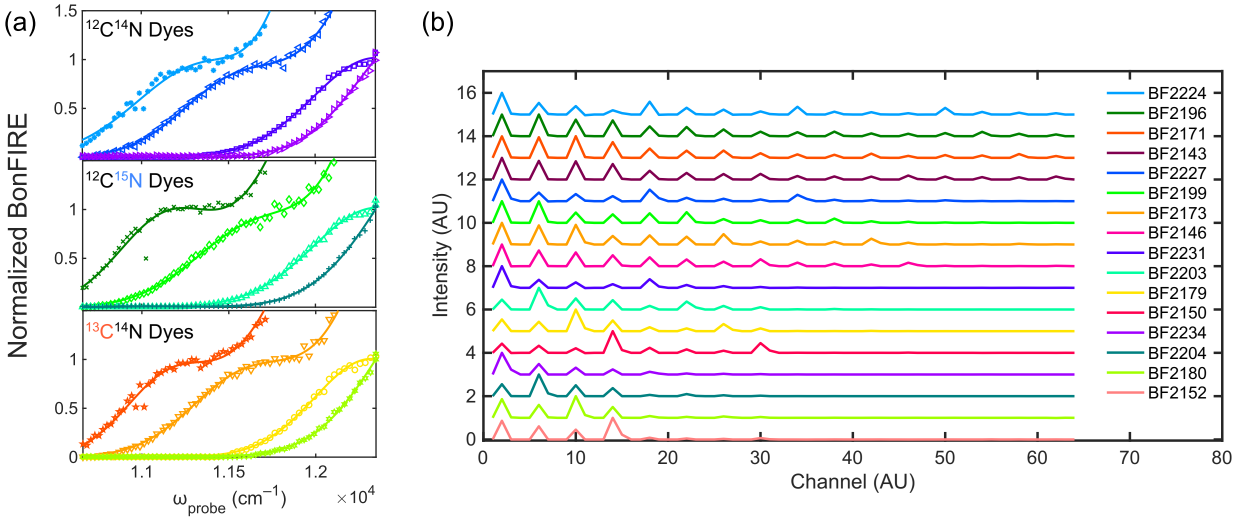

Figure S16. Probe reference spectra and unfolded hyperspectra of individual BonFIRE nitrile dyes. (a) Probe reference spectra of $^{12}C^{14}N$, $^{12}C^{15}N$, and $^{13}C^{14}N$ BF dyes ($^{13}C^{15}N$ dyes shown in **Fig. 4c**). (b) Unfolded hyperspectra of BF dyes in PS films. The hyperspectra are organized as three tiered groups (by t_D, ω_{IR}, and ω_{probe}) of four points.

We first attempted unmixing with the least absolute shrinkage and selection operator (LASSO), a regression method[27] that has recently demonstrated good success in unmixing of hyperspectral SRS images.[28] However, initial attempts with LASSO proved largely unsuccessful, with unmixing favoring the BF2234/2204/2180/2152 dyes over the others. We reasoned that

LASSO was struggling with our data for three main reasons. First, our spectra are extremely overlapped when presented as unfolded hyperspectra (**Fig. S16b**), as the spectra appear to have many peaks due to the presence of NDR-TPA (see **Section S3**); second, the distinguishing features between the spectra are too subtle on a linear scale for LASSO (a linear method) to accurately differentiate between the dyes; and third, our hyperspectra are sparsely sampled. As mentioned previously, our dataset here was intended to be the minimum possible dataset to distinguish between these dyes. Finer sampling in all three dimensions could lead to better LASSO performance.

However, we noted that the differentiating features in the reference spectra are visible by eye, implying that we should be able to achieve effective unmixing. Given that our dataset was rationally designed based on conditional logic (CL), we reasoned that similar if-then statements could be used to assist LASSO in unmixing, forming the basis of CL+LASSO. We also found that simple thresholds (i.e., SNR > 3) and binning (averaging with a range of neighboring pixels) were effective at suppressing crosstalk. Thus, we implemented CL+LASSO, as illustrated in **Fig. S17**.

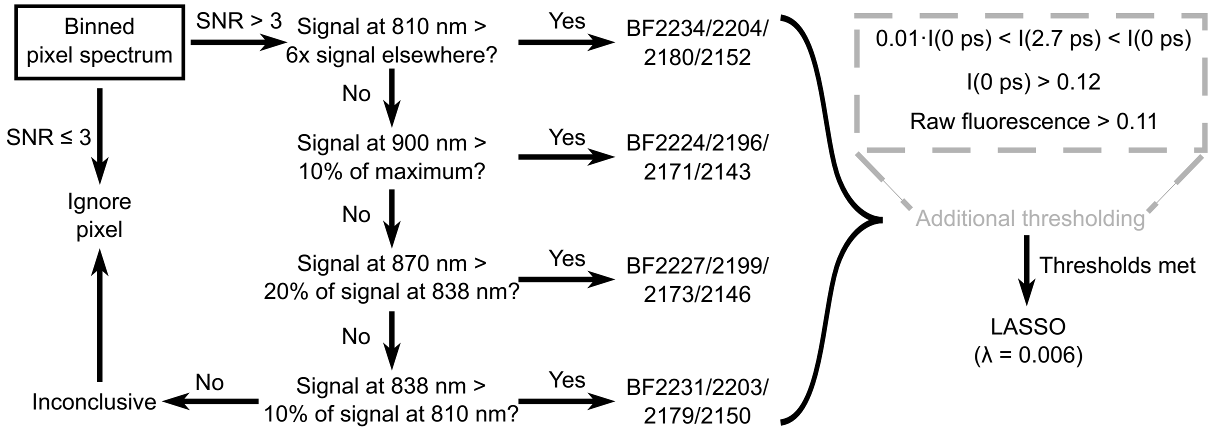

Figure S17. CL+LASSO implementation. This flowchart was implemented programmatically in MATLAB on a per-pixel basis, with ±5-neighbor binning.

As shown in the main text, CL+LASSO (**Fig. 4e**) yields robust unmixing. **Fig. S18** shows the individual components of the 16-color unmixed image, demonstrating that there is generally minimal crosstalk between the different dyes. Predictably, the best unmixing performance was obtained for dye films with the highest SNR (i.e., BF2179 and BF2203), and the films with the largest numbers of false positives had the lowest SNR (i.e., BF2146 and BF2173).

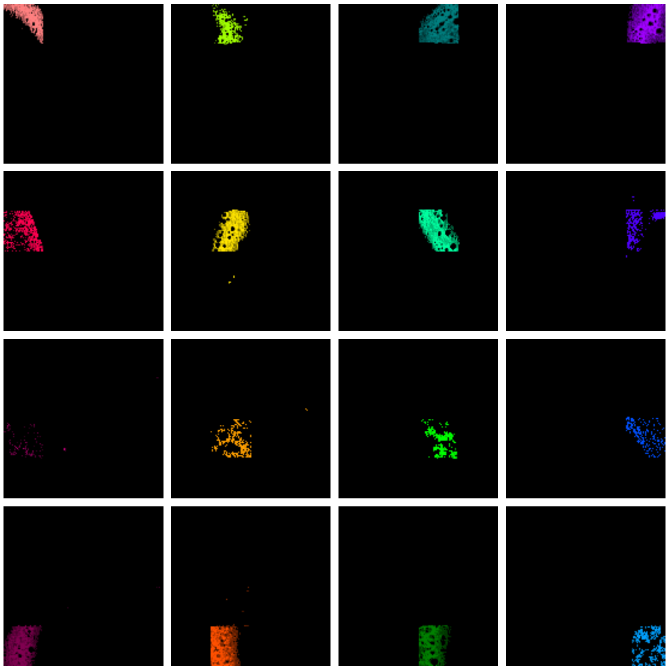

Figure S18. Unmixed 16-color component images from CL+LASSO. There is little to no crosstalk in most channels, with the lowest-SNR channels having the largest numbers of false positives.

It is important to note that the particular conditional logic used here is specific to our set of 16 nitrile dyes and their spectral differences in 2D-BonFIRE. However, given that the logic itself is based on the distinguishing spectral features present in the data, we reason that the concept of CL+LASSO should be generalizable beyond this dataset to any spectra with rationally identifiable differences.

S9. Vibrational lifetime imaging with sparse sampling.

As discussed in **Section S6** and in our previous work,[2] convolution fitting is the highest-accuracy method available for retrieving vibrational lifetimes from experimental data (errors <1%). However, the SNR requirements for convolution fitting can be quite stringent. In real-world imaging applications, such SNR is often not achievable, generally due to low analyte concentrations. Simultaneously, taking full hyperspectral images at many t_D positions can be prohibitively slow, since we generally acquire > 50 t_D points in a single lifetime measurement. To overcome these issues, we have previously demonstrated that fitting of a single exponential decay (without convolution) for t_D > 1.3 ps can be used to reproducibly obtain lifetime trends in the low-SNR regime, due to the reduced number of parameters in the fit.[2, 29] Most recently, we showed that a single exponential fit to the tail of a biexponential decay generally retrieves the weighted average lifetime ($\tau_{avg} = \frac{A_1\tau_1+A_2\tau_2}{A_1+A_2}$) with 8-13% error.[29] For monoexponential decays, the relative trend in lifetimes is retrieved by fitting with a single exponential without convolution,[1] but the absolute values systematically differ from those obtained by convolution.[2]

Here, for monoexponential decays, we attempt to bridge the gap between the highly accurate (but SNR-dependent) lifetimes obtained by convolution fitting and the SNR-independent (but less accurate) lifetimes obtained through non-convolution single exponential fitting. Using our published numerical simulation methods,[2] we find that non-convolution fitting is accurate for long lifetimes (95% accuracy for τ > 6.5 ps) and yields impressively consistent fitting, as evidenced by the near SNR-independence of the fitting accuracy (**Fig. S19a**). However, for lifetimes in the 0.5-2 ps range, non-convolution fitting errors range from 15%-80%, posing significant limits to quantitative analysis.

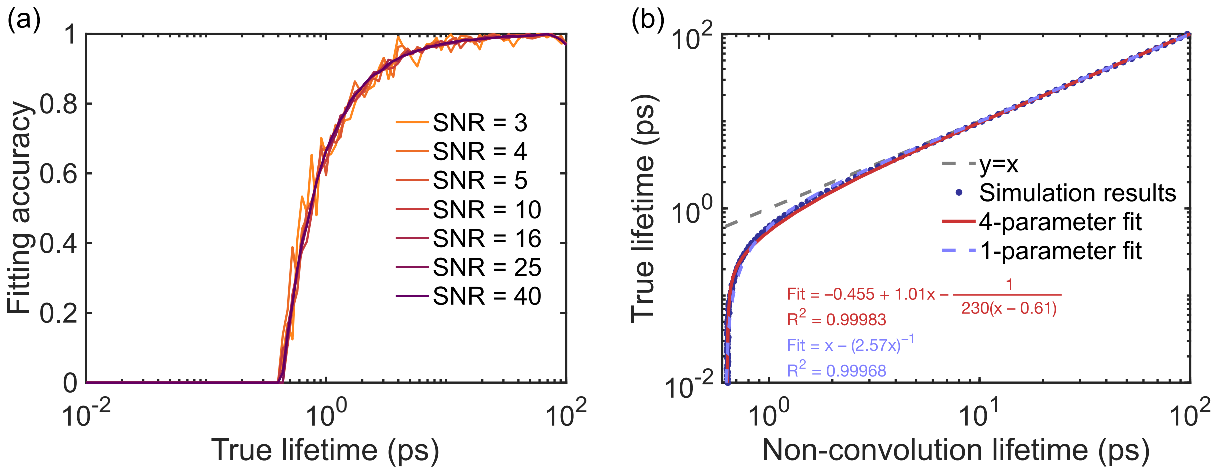

Figure S19. Non-convolution lifetime fitting. (a) Non-convolution fitting accuracy as a function of lifetime and SNR. (b) Relationship between true and non-convolution lifetimes. The trend is described well as a rational function.

Given the consistency of non-convolution fitting, we hypothesized that a calibration curve could be used to correct its inaccuracies for shorter lifetimes. By plotting the true lifetime as a function of the non-convolution lifetime (**Fig. S19b**), we observed a trend reminiscent of a rational function of the general form $y = a + bx + \frac{1}{c(x-d)}$, where $\lim_{x\to\infty} y = a + bx$ would account for higher accuracy at longer lifetimes and $\lim_{x\to d^+} y = \frac{1}{c(x-d)}$ accounts for the divergence at short lifetimes. Notably, the floor of $d \approx 0.6$ ps results from the shape of the Gaussian IRF, since the non-convolution fit sees the Gaussian as the dominant decay for short lifetimes. By fitting our data to a general rational function, we obtained a robust fit (red curve in **Fig. S19b**). We then wondered how many parameters could be removed from the fit, toward the ideal case of $\lim_{x\to\infty} y = x$, finding that remarkably similar fitting could be achieved with just one free parameter (lavender curve in **Fig. S19b**). Given its simplicity and accuracy, we used the 1-parameter model to obtain vibrational lifetimes for our vibrational lifetime multiplex imaging demonstration.

Supplementary Tables.

Table S1. Summary of 2D-BonFIRE data on Rh800.

ω_{FTIR} (cm^{-1})[a]	ω_{DFT} (cm^{-1})[b]	ω_{2D} (cm^{-1})[a]	$\omega_{max} - \omega_{probe}^{peak}$ (cm^{-1})[c]	$\lvert f_{FC}\rvert$[d]	τ_1 (ps)[e]	τ_2 (ps)[e]	A_1/A_2[e]
1204 ± 6	1196 [87]	1200 ± 10	1180 ± 200	0.010 ± 0.01	0.4 ± .8	9 ± 12	8 ± 15
1301 ± 3	1297 [98]	1303 ± 10	1320 ± 200	0.053 ± 0.01	0.7 ± .9	5.6 ± 3.4	2 ± 8
1361 ± 13	1362 [110]	1362 ± 10	1280 ± 200	0.008 ± 0.01	0.6 ± .7	8 ± 16	13 ± 22
1379 ± 5	1373 [112]	1379 ± 10	1280 ± 200	0.015 ± 0.01	1.0 ± .8	6.4 ± 2.9	7 ± 13
1432 ± 18	1448 [118]	1435 ± 10	1520 ± 200	0.067 ± 0.01	1.2 ± .7	6.2 ± 2.3	10 ± 14
1509 ± 6	1505 [129]	1506 ± 10	1480 ± 200	0.049 ± 0.01	0.9 ± .2	3.5 ± 2.3	7 ± 5
1544 ± 6	1544 [131]	1550 ± 10	1510 ± 200	0.027 ± 0.01	0.9 ± .4	6.2 ± 4.9	19 ± 16
1598 ± 7	1603 [134]	1595 ± 10	1410 ± 200	0.036[1]	1.3 ± .3	6.4 ± 3.2	9 ± 6
1648 ± 5	1652 [135]	1639 ± 10	1610 ± 200	0.040 ± 0.01	0.3 ± .3	4.3 ± 3.2	18 ± 18
2224 ± 5	2280 [136]	2230 ± 10	2140 ± 200	0.078[1]	1.2 ± .2	–	–
2710 ± 31	2815 [**118**+128]	2683 ± 10	1520 ± 200	0.088 ± 0.02	0.8 ± .4	3.9 ± 3.0	23 ± 17
2861 ± 31	2897 [117+**129**]	2854 ± 10	1450 ± 200	0.056 ± 0.02	0.2 ± .1	3.2 ± 1.2	36 ± 18
2946 ± 33	2925 [125+**129**]	2944 ± 10	1510 ± 200	0.034 ± 0.02	0.4 ± .6	4.1 ± 2.2	7 ± 11
n/a[f]	2926 [113+**134**]	2994 ± 10	1710 ± 200	n/a[f]	0.6 ± .4	3.0 ± 2.6	17 ± 20
3033 ± 14	3193 [**134**+135]	3039 ± 10	1770 ± 200	0.036 ± 0.02	0.4 ± .4	4.4 ± 7.0	16 ± 24

[a] Peak frequencies were obtained by least-squares fitting. Because the standard error of regression is likely to underestimate error, we use the peak half-width at half-maximum (for FTIR, ω_{FTIR}) and the IR pulse bandwidth (for 2D-BonFIRE, ω_{2D}) to estimate error.

[b] Harmonic DFT frequencies were scaled by 0.975. Mode assignments are listed in brackets. Our previously published anharmonic calculations[2] were used for the combination modes (the presumed FC-active mode is shown in bold).

[c] Values of $\omega_{max} - \omega_{probe}^{peak}$ tabulated here are obtained by fitting probe spectra and thus differ from the values plotted in the main text figures. Error is estimated to be 200 cm^{-1}.

[d] Relative values of $|f_{FC}|$ are obtained by $\sqrt{I_{2D}(\omega_{IR}, \omega_{probe}) / \left[\sigma_{FTIR}(\omega_{IR})\sigma_{UV-vis}(\omega_{probe} + (\omega_{max} - \omega_{probe}^{peak}))\right]}$, which assumes identical σ between BonFIRE and FTIR/UV-vis. Absolute values are obtained by reference to previously published values of f_{FC} obtained by quantum mechanical methods.[1] Error is estimated to be 0.01 for fingerprint modes and 0.02 for combination modes due to lower SNR.

[e] Lifetime fit values are reported as mean (standard deviation). Values of τ_1 < 0.5 ps accompanied by A_1/A_2 >> 10 generally indicate that our SNR is insufficient to resolve both components of the biexponential decay (biexponential decays with limited SNR appear as monoexponential decays). It should be noted that far better precision is obtained by replicate measurements at single frequencies rather than broadband scanning, as previously demonstrated.[2]

[f] A clear peak was not visible in FTIR near 2990 cm^{-1}. Thus, ω_{FTIR} and σ_{FTIR} (and therefore $|f_{FC}|$) could not be obtained for this mode.

Table S2. Summary of 2D-BonFIRE frequency-domain data across all molecules.

Dye	ω_{IR} (cm^{-1})[a]	$\omega_{max} - \omega_{probe}^{peak}$ (cm^{-1})[b]	Dye	ω_{IR} (cm^{-1})[a]	$\omega_{max} - \omega_{probe}^{peak}$ (cm^{-1})[b]
Rh800[c]	1200	1180	ATTO725	1594	1340
Rh800	1303	1320	ATTO725	2226	2130
Rh800	1362	1280	ATTO725	2714	1400
Rh800	1379	1280	Cy5.5	1477	1520
Rh800	1435	1520	BF2224	2223.9	2250
Rh800	1506	1480	BF2227	2226.9	2210
Rh800	1550	1510	BF2231	2231.2	2310
Rh800	1595	1410	BF2234	2234.3	2000
Rh800	1639	1610	BF2196	2196.1	2200
Rh800	2230	2140	BF2199	2198.5	2230
Rh800	2683	1520	BF2203	2204.2	2340
Rh800	2854	1450	BF2204	2204.4	2020
Rh800	2944	1510	BF2171	2171.3	2280
Rh800	2994	1710	BF2173	2173.1	2180
Rh800	3039	1770	BF2179	2179.6	2340
ATTO665	1596	1580	BF2180	2180.1	2040
ATTO665	2848	1480	BF2143	2142.9	2200
ATTO680	1597	1540	BF2146	2146	2180
ATTO680	2713	1580	BF2150	2150.4	2250
ATTO680	2773	1650	BF2152	2151.7	2070

The data in this table are also plotted in **Fig. 2f**. MgPc and ICG-d_7 were excluded due to insufficient spectral coverage and SNR, respectively.

[a] As with **Table S1**, we take 10 cm^{-1} as a conservative estimate of our error in ω_{IR}, though we note for the BF dyes that the error appears closer to 1 cm^{-1} for this dataset.

[b] As with **Table S1**, error in $\omega_{max} - \omega_{probe}^{peak}$ is estimated to be 200 cm^{-1}.

[c] Values for Rh800 are reproduced from **Table S1**.

References

1. H. Wang, D. Lee, Y. Cao, X. Bi, J. Du, K. Miao and L. Wei, Bond-selective fluorescence imaging with single-molecule sensitivity, *Nat. Photonics*, 2023, **17**, 846-855.
2. P. A. Kocheril, H. Wang, D. Lee, N. Naji and L. Wei, Nitrile Vibrational Lifetimes as Probes of Local Electric Fields, *J. Phys. Chem. Lett.*, 2024, **15**, 5306-5314.
3. L. Wei, Z. Chen, L. Shi, R. Long, A. V. Anzalone, L. Zhang, F. Hu, R. Yuste, V. W. Cornish and W. Min, Super-multiplex vibrational imaging, *Nature*, 2017, **544**, 465-470.
4. Y. Miao, N. Qian, L. Shi, F. Hu and W. Min, 9-Cyanopyronin probe palette for super-multiplexed vibrational imaging, *Nat. Commun.*, 2021, **12**, 4518.
5. F. Ghani, J. Kristen and H. Riegler, Solubility Properties of Unsubstituted Metal Phthalocyanines in Different Types of Solvents, *J. Chem. Eng. Data*, 2012, **57**, 439-449.
6. A. Laubereau, S. F. Fischer, K. Spanner and W. Kaiser, Vibrational population lifetimes of polyatomic molecules in liquids, *Chem. Phys.*, 1978, **31**, 335-344.
7. M. Wojdyr, Fityk: a general-purpose peak fitting program, *J. Appl. Crystallogr.*, 2010, **43**, 1126-1128.
8. N. H. Gottfried, A. Seilmeier and W. Kaiser, Transient internal temperature of anthracene after picosecond infrared excitation, *Chem. Phys. Lett.*, 1984, **111**, 326-332.
9. M. D. Hanwell, D. E. Curtis, D. C. Lonie, T. Vandermeersch, E. Zurek and G. R. Hutchison, Avogadro: an advanced semantic chemical editor, visualization, and analysis platform, *J. Cheminform.*, 2012, **4**, 17.
10. M. J. Frisch, G. W. Trucks, H. B. Schlegel, G. E. Scuseria, M. A. Robb, J. R. Cheeseman, G. Scalmani, V. Barone, G. A. Petersson, H. Nakatsuji, X. Li, M. Caricato, A. V. Marenich, J. Bloino, B. G. Janesko, R. Gomperts, B. Mennucci, H. P. Hratchian, J. V. Ortiz, A. F. Izmaylov, J. L. Sonnenberg, D. Williams-Young, F. Ding, F. Lipparini, F. Egidi, J. Goings, B. Peng, A. Petrone, T. Henderson, D. Ranasinghe, V. G. Zakrzewski, J. Gao, N. Rega, G. Zheng, W. Liang, M. Hada, M. Ehara, K. Toyota, R. Fukuda, J. Hasegawa, M. Ishida, T. Nakajima, Y. Honda, O. Kitao, H. Nakai, T. Vreven, K. Throssell, J. A. J. Montgomery, J. E. Peralta, F. Ogliaro, M. J. Bearpark, J. J. Heyd, E. N. Brothers, K. N. Kudin, V. N. Staroverov, T. A. Keith, R. Kobayashi, J. Normand, K. Raghavachari, A. P. Rendell, J. C. Burant, S. S. Iyengar, J. Tomasi, M. Cossi, J. M. Millam, M. Klene, C. Adamo, R. Cammi, J. W. Ochterski, R. L. Martin, K. Morokuma, O. Farkas, J. B. Foresman and D. J. Fox, Gaussian 16, Revision B.01, 2016.
11. T. Tamura, P. C. McCann, R. Nishiyama, K. Hiramatsu and K. Goda, Fluorescence-Encoded Time-Domain Coherent Raman Spectroscopy in the Visible Range, *J. Phys. Chem. Lett.*, 2024, **15**, 4940-4947.
12. A. Seilmeier, P. O. J. Scherer and W. Kaiser, Ultrafast energy dissipation in solutions measured by a molecular thermometer, *Chem. Phys. Lett.*, 1984, **105**, 140-146.
13. J. M. Hales, D. J. Hagan, E. W. Van Stryland, K. J. Schafer, A. R. Morales, K. D. Belfield, P. Pacher, O. Kwon, E. Zojer and J. L. Bredas, Resonant enhancement of two-photon absorption in substituted fluorene molecules, *J. Chem. Phys.*, 2004, **121**, 3152-3160.
14. J. P. Maier, A. Seilmeier and W. Kaiser, Population lifetime of CH-stretching modes in medium-size molecules, *Chem. Phys. Lett.*, 1980, **70**, 591-596.

15. F. Wondrazek, A. Seilmeier and W. Kaiser, Ultrafast intramolecular redistribution and intermolecular relaxation of vibrational energy in large molecules, *Chem. Phys. Lett.*, 1984, **104**, 121-128.
16. L. Whaley-Mayda, A. Guha and A. Tokmakoff, Multimode vibrational dynamics and orientational effects in fluorescence-encoded infrared spectroscopy. II. Analysis of early-time signals, *J. Chem. Phys.*, 2023, **159**, 194202.
17. M. Sakai, Vibrational Energy Relaxation Process of the 7-Azaindole Dimer in Gas Phase and Solution, *Bunko Kenkyu*, 2005, **54**, 163-169.
18. P. Geerlings, D. Berckmans and H. P. Figeys, The influence of electrical and mechanical anharmonicity on the vibrational transition moments of diatomic and polyatomic molecules, *J. Mol. Struct.*, 1979, **57**, 283-297.
19. D. Loru, W. Sun, H. Nootebos, A. L. Steber, P. Ferrari and M. Schnell, Probing the structure and dynamics of the heterocyclic PAH xanthene and its water complexes with infrared and microwave spectroscopy, *Phys. Chem. Chem. Phys.*, 2024, **26**, 25341-25351.
20. H. Salzmann, A. B. McCoy and J. M. Weber, Infrared Spectrum of the Pyrene Anion in the CH Stretching Region, *J. Phys. Chem. A*, 2024, **128**, 4225-4232.
21. M. Majoube and M. Henry, Fourier transform Raman and infrared and surface-enhanced Raman spectra for rhodamine 6G, *Spectrochim. Acta A Mol. Spectrosc.*, 1991, **47**, 1459-1466.
22. A. Seilmeier and W. Kaiser, in *Ultrashort Laser Pulses and Applications*, ed. W. Kaiser, Springer Berlin, Heidelberg, 1988, ch. 7, pp. 279-317.
23. P. A. Kocheril, D. Lee, N. Naji, R. S. Chadha, R. E. Leighton, H. Wang and L. Wei, Single-molecule Vibrational Thermometry, *ChemRxiv*, 2025, **83zc3**.
24. A. Seilmeier, J. P. Maier, F. Wondrazek and W. Kaiser, Relaxation of vibrational energy of polyatomic molecules incorporated in a plastic matrix, *J. Phys. Chem.*, 1986, **90**, 104-108.
25. X. Wen, W. A. Tolbert and D. D. Dlott, Multiphonon up-pumping and molecular hot spots in superheated polymers studied by ultrafast optical calorimetry, *Chem. Phys. Lett.*, 1992, **192**, 315-320.
26. X. Hong, S. Chen and D. D. Dlott, Ultrafast Mode-Specific Intermolecular Vibrational Energy Transfer to Liquid Nitromethane, *J. Phys. Chem.*, 1995, **99**, 9102-9109.
27. R. Tibshirani, Regression Shrinkage and Selection via the Lasso, *J. R. Statist. Soc. B*, 1996, **58**, 267-288.
28. Y. Tan, H. Lin and J.-X. Cheng, Profiling single cancer cell metabolism via high-content SRS imaging with chemical sparsity, *Sci. Adv.*, 2023, **9**, eadg6061.
29. H. Wang, P. A. Kocheril, Z. Yang, D. Lee, N. Naji, J. Du, L. E. Lin and L. Wei, Room-Temperature Single-Molecule Infrared Imaging and Spectroscopy through Bond-Selective Fluorescence, *Angew. Chem. Int. Ed.*, 2024, **63**, e202413647.
